# SNP Marker Discovery in Koala TLR Genes

**DOI:** 10.1371/journal.pone.0121068

**Published:** 2015-03-23

**Authors:** Jian Cui, Greta J. Frankham, Rebecca N. Johnson, Adam Polkinghorne, Peter Timms, Denis O’Meally, Yuanyuan Cheng, Katherine Belov

**Affiliations:** 1 Faculty of Veterinary Science, University of Sydney, Sydney, NSW, Australia; 2 Australian Museum Research Institute, Australian Museum, Sydney, NSW, Australia; 3 Faculty of Science, Health, Education and Engineering, University of the Sunshine Coast, Sippy Downs, Queensland, Australia; CSIRO, AUSTRALIA

## Abstract

Toll-like receptors (TLRs) play a crucial role in the early defence against invading pathogens, yet our understanding of TLRs in marsupial immunity is limited. Here, we describe the characterisation of nine TLRs from a koala immune tissue transcriptome and one TLR from a draft sequence of the koala genome and the subsequent development of an assay to study genetic diversity in these genes. We surveyed genetic diversity in 20 koalas from New South Wales, Australia and showed that one gene, *TLR10* is monomorphic, while the other nine TLR genes have between two and 12 alleles. 40 SNPs (16 non-synonymous) were identified across the ten TLR genes. These markers provide a springboard to future studies on innate immunity in the koala, a species under threat from two major infectious diseases.

## Introduction

The koala (*Phascolarctos cinereus*) is an arboreal herbivorous marsupial which was widespread across eastern Australia until the end of the 19^th^ century, when populations have suffered declines due to the fur trade, habitat degradation and disease [1]. The koala is distributed from Queensland, New South Wales (NSW) and Victoria and translocated to islands off the south coast of Australia and to South Australia [2]. The Queensland, NSW and Australian Capital Territory populations are listed as “vulnerable” under the federal government *Environmental Protection and Biodiversity Conservation Act* 1999. Genetic diversity in koalas is extremely low on the introduced islands and in Southern Australia due to founder effects [3]. Levels of diversity are higher in NSW and Queensland [3]. North-eastern koalas have twice as much diversity as south-eastern koalas (A = 11.5+/−1.4 vs A = 5.3+/−1), and microsatellite variability is comparable to that seen in other wild species [3,4]. Thirty-one mitochondrial haplotypes from the hypervariable Control Region (D-loop), have been characterized across the Australian east coast distribution [5,6], including 9 novel haplotypes recently characterized from NSW (unpublished data, Australian Museum). Mitochondrial DNA haplotype diversity within south-eastern Queensland and north-eastern NSW populations was much higher than that in Victoria and South Australian island populations, which contained only a single mitochondrial haplotype [5]. Diversity in key immune genes belonging to the Major Histocompatibility Complex (MHC) in NSW and Queensland koalas is also robust [7], and is on par with that of New Zealand brushtail possums (*Trichosurus vulpecula*) [7,8].

Many koala populations are currently threatened by habitat loss and vehicular injuries [1] as well as infections by koala retrovirus (KoRV), which is associated with immune suppression and lymphoma and leukemia [9] and by *Chlamydia pecorum and C*. *pneumoniae* [10], obligate intracellular bacterial pathogens that cause debilitating ocular and reproductive tract disease [11]. In wild populations, chlamydial infection rates range from 20% to 100% [11]. However, depending on the populations, the level of clinical disease in association with infection has been noted to vary. This anecdotal evidence supported by studies monitoring *C*. *pecorum* shedding in individual koalas with and without clinical disease [12], would suggest that infection alone is not the primary determinant of chlamydial disease development in this host. Indeed, while studies continue to emerge that key genetic differences may exist in the infecting chlamydial strain in koalas [13] studies in other hosts suggest that the host’s immune response is key to the eventual outcome of the infection [14]. KoRV, on the other hand, is in the process of endogenising into the koala’s genome. While the direct relationship of KoRV to diseases such as leukemia and lymphoma are yet to be proven, it is widely assumed that the integration of KoRV sequences into the koala genome has affected the koala’s immune responsiveness to KoRV and potentially chlamydial infections [15].

Toll-like receptors (TLRs), encoded by a range of TLR genes, are key components of the innate immune response. TLRs are the first receptors to interact with invading microorganisms by recognising pathogen-associated molecular patterns (PAMPs) on a wide spectrum of pathogens [16]. TLRs are encoded by a large gene family, with 10 TLR homologs (TLR1–10) characterised in human (*Homo sapiens*; TLR1–10), cow (*Bos taurus*) and pig (*Sus domesticus*), 12 in house mouse (*Mus musculus*) [16–18] and 10 in gray short-tailed opossum (*Monodelphis domestica*) [19] and Tasmanian devil (*Sarcophilus harrisii*) [20]. The TLR molecules contain three domains; a large extracellular domain consisting of 18–30 Leucine-Rich Repeats (LRRs), a transmembrane domain and an intracellular Toll/interleukin I (TIR) domain. The extracellular domain forms a horseshoe shape, and it can recognize bacteria, fungi, parasites and viruses. TLRs can be sub-divided based on their functional roles into viral and non-viral. Viral TLRs are expressed in the cell, and include TLR3, TLR7, TLR8 and TLR9. They can recognize dsRNA and DNA viruses (TLR3) [21], ssRNA (TLR7 and TLR8) [22] and unmethylated CpG-containing DNA, which is commonly found in the genomes of DNA viruses (TLR9) [23]. Non-viral TLRs are expressed on cell surface and can respond to lipopeptide from bacteria and parasites (TLR1, TLR2, TLR6 and TLR10) [24,25], lipopolysaccharides (LPS) from Gram-negative bacteria (TLR4) [26], flagellins (TLR5) [25] and bacterial 23S ribosomal RNA (TLR13) [27]. Although most immunological studies have been limited to mouse models, there is strong evidence to suggest that TLR2 and/or TLR4 activation and signaling has a strong influence on the clearance of the development of immunopathological sequelae as a result of chlamydial infection [28]. Interestingly, recent studies in humans have revealed that genetic Variants in the TLR1 and TLR4 genes may increase inflammation and are associated with risk of chlamydial infection and development of pelvic inflammatory disease [29], raising questions over whether genetic variation at these loci may serve as a biomarker of chlamydial infection and disease in other species as well.

Genomics technologies have facilitated rapid elucidation of the basic architecture of the koala’s immune system. Recent studies have described the characterisation of MHC class I and II [7,8,30], interleukins [31–33], interferon gamma, T cell markers and other immune genes [34]. In an effort to provide more tools to understand the role of TLRs in the koala response to infectious diseases, in the current study, we describe nine TLR-encoding genes in the koala and the development of a series of molecular markers that may be applied to study TLR genetic diversity in koala populations with different chlamydial infection outcomes.

## Material and Methods

### Koala transcriptome dataset

The koala transcriptome dataset consists of four cDNA libraries which were established from immune related tissues including liver, lymph node, spleen and bone marrow [35]. All tissues are from a single male koala obtained from the Australia Zoo Wildlife Hospital. All cDNA libraries were deposited in the Sequence Read Archive at NCBI [31] with the accession number SRR1106690, SRR1106707, SRR1121764, SRR1122141 for bone marrow, lymph node, liver and spleen libraries, respectively. The koala genome is published as a marker paper [36] and was made available to us for this project.

### Searching strategy

Nine koala TLR genes were identified by searching all 4 koala cDNA libraries [35] using BLAST with human (*Homo sapiens*) and mouse (*Mus musculus*) TLR coding sequences. Koala TLR13 was obtained by searching the draft koala genome using BLAST using a Tasmanian devil (*Sarcophilus harrisii*) TLR13 nucleotide coding sequence [20]. Phylogenetic relationships between koala TLR genes and their homologs in eight other species, including three eutherians, human, cow and mouse, and two marsupial species, gray short-tailed opossum and Tasmanian devil [20], one bird species (chicken, *Gallus gallus*), one amphibian (western clawed frog, *Xenopus tropicalis*) and one fish species (zebrafish, *Danio rerio*) were analysed in MEGA 5 [37] using the Neighbour joining method [38] with 1000 bootstrap replicates to infer the level of confidence on the phylogeny. GenBank and Ensembl accession numbers of sequences are listed in S1 Table.

### Analysis of TLR diversity in 20 wild koalas

Genetically diverse koalas were selected in an attempt to maximize the TLR gene diversity discovered in this study. Control Region mitochondrial diversity was used as a proxy measure to choose 20 genetically diverse animals, representing six koala mitochondrial DNA (mtDNA) Control Region haplotypes (Australian Museum, unpublished data). These koalas were all from New South Wales ranging from Narrandera (southern NSW) to Kyogle (northern NSW). Koala samples were either collected by the Port Macquarie Koala Hospital when animals were brought in for veterinary care or from the Australian Museum Tissue Collection. Australian Museum registration numbers are provided in S2 Table. For mtDNA PCR protocols and conditions see Frankham et al 2014 [39].

Gene specific primers (Table 1) were designed using Oligo6 [40]. Primers for TLR2, TLR5, TLR6, TLR7, TLR8, TLR9 and TLR13 were designed at both ends of the coding region, amplifying the LRRs, transmembrane and cytoplasmic domains since the coding sequences of these genes are encoded within a single exon. The target fragments of TLR3, TLR4 and TLR10 contain partial LRR region and partial cytoplasmic region and include the peptide-binding region for each gene. The coding sequences of these three genes have multiple exons. PCR amplifications were carried out in a Bio-Rad MJ Mini Personal Thermal Cycler in 25μl reactions containing 1× high-fidelity buffer (Invitrogen, Mulgrave, Australia) that consists of 60 mM Tris-HCl (pH8.9) and 18 mM (NH_4_)_2_SO_4_, 0.2 mM each dNTP, 2.0 mM MgSO_4_, 0.5 uM each forward and reverse primers, 1.5U of Platinum *Taq* DNA Polymerase High Fidelity (Invitrogen, Mulgrave, Australia) and approximately 30 ng template DNA. The general cycle conditions were initial denaturation at 94°C for 2 min, followed by 35 cycles of 30 s denaturation at 94°C, 30 s annealing at 55–66°C (Table 1), and 1–3 min extension at 68°C and a final extension at 68°C for 10 min. A negative control without DNA was included in all PCR reactions. PCR products were isolated on a 1.2% agarose gel, using EasyLadder I (Bioline, Alexandria, Australia) as a size marker, and purified with QIAEX II Gel Extraction Kit (QIAGEN, Chadstone Centre, Australia). PCR products were inserted into pGEM-T Easy Vector System I (Promega, Alexandria, Australia), and then transformed into JM109 High-Efficiency Competent Cells (Promega, Alexandria, Australia). Eight positive clones were picked for each PCR sample to make sure we captured both alleles from each locus within each individual. The plasmids were extracted using DirectPrep 96 MiniPrep Kit (QIAGEN, Chadstone Centre, Australia) and sequenced with T7 forward and SP6 reverse primers at Australian Genome Research Facility (AGRF, Westmead, Australia).

**Table 1:** PCR primers for nine TLR genes in the koala.

**Genes**	**CDS length(bp)**	**Target fragment(bp)**	**Primer sequence (5'-3')**		**T**_a_ **(°C)**
TLR2	2350	2290(23–2312)	F	ATGAGACATGCCATGTGGACAG	66
			R	AGCTCTCAAACTGACCCAAAACAAT	
TLR3	2718	1502(926–2427)	F	GAATGGCTTGTGTTGGAGTATA	56
			R	CAAAAATAATCTTCCTGCTCCT	
TLR4	2499	2036(315–2350)	F	AGGGCTTGTATAATCTCTCCACTTT	62
			R	ATAGGTGTTTCGACGTAGCAGAC	
TLR5	2580	2468(27–2494)	F	GCCACCTCCTTGCATTTCTCTT	63
			R	TTTTGGTGAGTATGCATTGAGAGAGTT	
TLR1/6like	2418	2334(24–2357)	F	ATGACAGTGACCCTTAAGGATGT	53
			R	TCATATTAAATGCTGCTTTGATGTTA	
TLR7	3141	3065(23–3087)	F	ATGCGAGAATTATACAGACCGT	65
			R	CTGTGTCATTGTCTGTGGCTAT	
TLR8	3126	3035(43–3077)	F	AAGTTTTACCTGTTTGTACGTTCTG	60
			R	GTTGTACCGGCTATCATTGTCT	
TLR9	3120	2962(38–2999)	F	GGTTTCTCGTGCTTGAGGTTTC	66
			R	GTGAGGCCAGAAGAGGACAGTC	
TLR10	2436	1606(741–2346)	F	GTCCAACAGGCTCTAAAACTTC	60
			R	ACTTGTTCATCTTTCCCAATTC	
TLR13	2862	2196(215–2410)	F	ATCACTCACCTCAACCTTACAC	57
			R	GCTCCTTATATACCCACTGCTC	

Sequences were quality-checked with Sequencher 4.9 and aligned in BioEdit v7.2.3 [41]. To minimise sequence artefacts from PCR, cloning and sequencing, a sequence variant was considered a real TLR allele when identified in multiple PCR amplifications from each individual.

The overall rate of synonymous substitution per synonymous site (*d*_*S*_) and non-synonymous substitutions per non-synonymous sits (*d*_*N*_) in each gene were conducted using the Nei-Gojobori method with Jukes-Cantor adjustment in MEGA 5 [37]. Codon-based Z-tests of selection were performed with 5000 bootstrap replications to generate the standard error.

## Result and Discussion

**Fig 1:**
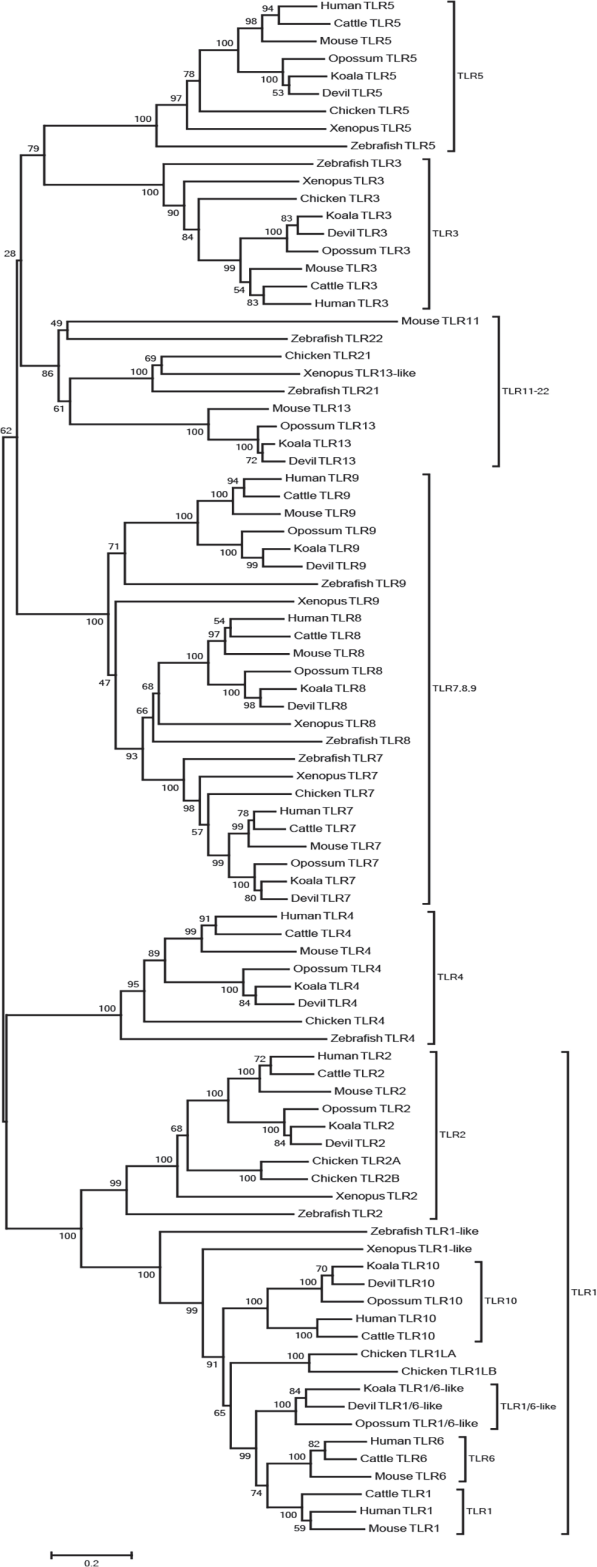
Neighbour-joining phylogenetic analysis of TLRs. Ten TLR amino acid sequences of the koala compared to TLRs in eight species, including three eutherians (human, cattle and house mouse), two marsupials (Tasmanian devil and gray short-tailed opossum), chicken, Xenopus and zebrafish. The bootstrap values are displayed at each branch point.

We identified nine koala TLRs with clear eutherian orthologs (TLR2, TLR3, TLR4, TLR5, TLR7, TLR8, TLR9, TLR10 and TLR13). The evolutionary relationships of these genes are depicted in Fig. 1, and resemble those previously described by Roach et al. 2005. TLRs are present across a wide range of taxonomic groups, including insects [42,43], fish [44,45], amphibian [46], birds [47,48] and mammals [16,49]. TLRs from each family have similar functions across species [50,51]. The koala TLR genes shared on average 64% amino acid identify to their human counterparts, with TLR3 and TLR7 the highest at 73%, and TLR4 the lowest at 53%. The transcripts described here contained complete coding sequences, and ranged in length from 2350 (TLR2) to 3141bp (TLR7), similar to the human TLRs, which ranged from 2352 (TLR2) to 3147bp (TLR7). The primers we designed to study diversity amplified between 1502 and 3065bp (details provided in Table 1).

Based on the well characterized human TLR sequences, the koala TLR genes contained all of the key functionally conserved residues in the extracellular domain [25] S2–S11 Figs. The leucine-rich motif in each LRR was conserved. Residues 1–10 in every LRR motif were present in all LRRs and are predicted to form a β-strand within each LRR. The residues which follow residue 10 are variable among TLRs, and this variability is usually associated with pathogen binding S12–S21 Figs. [25,52–56].

The phylogenetic tree shown in Fig. 1 highlights that vertebrate TLRs can be divided into six subfamilies which include the TLR1 family (including TLR1, TLR6, TLR10 and TLR2), TLR3, TLR4, TLR5, the TLR7 family (including TLR7, TLR8 and TLR9) and the TLR11 family (including TLR11–22). All vertebrate species appear to have at least one copy of a gene from each subfamily. As expected, the koala TLR2, TLR3, TLR4, TLR5, TLR7, TLR8 TLR9 TLR10 and TLR13 each had a single clear ortholog in the other marsupial and eutherian species, so we were able to annotate koala TLRs confidently. The koala sequences have been deposited to GenBank and have been assigned the GenBank accession numbers, S3 Table. A single gene, which we designate TLR1/6-like due to its location at the base of the eutherian TLR1 and TLR6 clades (Fig. 1) was also found in the transcriptome assembly. This gene, while not reported in humans or other eutherians, has been found in opossum [19] and the Tasmanian devil [20] and its emergence appears to predate the duplication of TLR1 and TLR6 in eutherians [19]. It shares 58.5% amino acid identity with human TLR1 and 60.2% amino acid identify with human TLR6.

The phylogenetic tree allows us to speculate about the evolutionary history of the TLR1 gene family. A single TLR1-like gene and a single TLR2 gene are seen in zebrafish and Xenopus [19]. The chicken genome contains two copies of TLR1, which duplicated relatively recently, as depicted by the short branch lengths, as well as two copies of TLR2 genes. It does not contain TLR10. It appears that the ancestral avian TLR1, duplicated to give rise to TLR10 and TLR1/6-like in mammals. TLR1/6-like is located adjacent to TLR10 in the koala, Tasmanian devil and opossum genomes and is found in present day marsupials. TLR1/6-like then went on to duplicate to give rise to the TLR1 and TLR6 families, which are seen in present day eutherians. The mammals all retained TLR10 and TLR2.

The TLR genes evolve independently in different lineages in response to species-specific pathogens [57]. Genes in the TLR1 subfamily form homo or heterodimers with each other and with TLR2, indicating redundancy in pathogen recognition. For instance, the human TLR2/TLR1 heterodimer responds to microbial triacyl lipoproteins [58], and TLR2/TLR6 responds to diacyl lipopeptides [59], however, mutation of the F343 and F365 residues in TLR6 allows the TLR2/TLR6 heterodimer to respond to triacyl lipopeptides [59]. Similarly, heterodimers of human TLR1/TLR2 and TLR2/TLR10 can recognize the same pathogens [60]. On the other hand, in chickens, heterodimers of any TLR1/TLR2 combination can respond to diacyl and triacyl lipopeptides [61]. We therefore predict that marsupial TLR1/6-like will also be able to form homo and heterodimers with other members of the TLR1 family and respond to diacyl and triacyl lipopeptides.

Genetic polymorphisms were detected in all koala TLR genes, except TLR10. In the nine polymorphic genes, the number of alleles per gene ranged from 2–12, and the number of SNPs per gene between 1–8 (Table 2). In this study, TLR4 showed the highest level of genetic variability, with 12 alleles containing 8 SNPs. It is also the most polymorphic TLR in humans, cattle, pigs and robins [62–65]. A total of 40 SNPs were identified across all loci, all of which were biallelic and 16 were non-synonymous (Table 2). 13 of the non-synonymous SNPs were located in the extracellular domain, while the others in the intracellular domain. For each individual we have provided a summary of TLR genotypes and mitochondrial genotypes in S4 Table.

**Table 2:** Polymorphisms in koala TLRs.

**Gene**	**Observe haplotypes**	**syn:nsyn**	**SNPs**	**Amino acid**
TLR2	4	3:0	213: C/T	Asp
			834: G/A	Leu
			1665: A/C	Thr
TLR3	4	3:1	471: T/C	Thy
			1059: T/C	Leu
			1236: C/T	Val
			1339: G/A	Asp/Asn
TLR4	12	5:3	474: C/T	Ile
			576: T/C	Asn
			867: A/G	Thr
			959: C/A	Ser/Tyr
			1458: A/G	Gln
			1543 C/G	Leu/Val
			1660: A/G	Met/Val
			1812: C/T	Tyr
TLR5	8	3:4	509: A/G	Gln/Arg
			528: A/G	Val
			589: A/G	Asn/Asp
			864: C/T	His
			1452: A/G	Ser
			1723: C/G	Gln/Glu
			2117: A/G	His/Arg
TLR1/6like	2	1:0	384: T	Thr
TLR7	4	3:2	102: T/C	Ser
			385: G/A	Glu/Lys
			807: C/A	Ile
			1970: A/G	Glu/Gly
			2439: C/T	Ser
TLR8	5	2:2	909: T/C	Phe
			1319: C/A	Thr/Lys
			1352: T/C	Met/Thr
			1935: A/G	Pro
TLR9	10	4:3	136: A/G	Thr/Ala
			701: C/T	Ala/Val
			1158: C/T	Leu
			1917: T/C	Pro
			2033: A/G	His/Arg
			2043: G/A	Thr
			2709: G/A	Arg
TLR13	2	0:1	2122: C/A	Arg/Ser

The level of TLR diversity that was observed in koalas in this study is comparable to that observed in other species (Table 3). For instance, 73 alleles have been identified at TLR5 in 158 humans from Africa, Europe and East-Asia [66]. A study on 259 pigs from six populations identified 16 SNPs within the TLR4 exon 3. To make our comparison more relevant, we have compared the koala results with those 56 outbred pigs, rather than incorporating inbred populations [67]. Grueber et al. 2012 studied a bottlenecked population of 24 New Zealand Robins from an isolated island population, and observed a range of 1–5 alleles within all TLRs coding sequences [65] (Table 3). The koala samples used here were selected to maximize genetic diversity, but it is important to note that additional alleles are likely to be found if more samples were analysed, particularly if samples from additional geographic regions were analysed.

**Table 3:** Comparison of TLR polymorphisms between koala, New Zealand Robin, pig and human.

**Species**	**Genes**	**Samples**	**SNPs**	**Alleles**	**References**
Koala, *Phascolarctos cinereus*	TLR2	20	3	4	This study
	TLR3		4	4	
	TLR4		8	12	
	TLR5		7	8	
	TLR1/6like		1	2	
	TLR7		5	4	
	TLR8		4	5	
	TLR9		7	10	
	TLR10		0	1	
	TLR13		1	2	
New Zealand Robin, *Petroica*	TLR2A	17–24	1	2	[65]
*australis rakiura*	TLR2B		5	3	
	TLR3		0	1	
	TLR4		5	5	
	TLR5		2	3	
	TLR7		2	2	
Pig, *Sus scrofa*	TLR4	56	16	23	[67]
Human, *Homo sapiens*	TLR1	158	59	52	[66]
	TLR2		24	23	
	TLR3		41	55	
	TLR4		66	59	
	TLR5		73	61	
	TLR6		44	36	
	TLR7		26	31	
	TLR8		33	46	
	TLR9		27	30	
	TLR10		64	55	

By comparing rates of synonymous (d_S_) and non-synonymous (d_N_) substitutions [68] we found that d_S_ is higher than d_N_ in all nine koala TLR genes characterized here. No non-synonymous substitutions were observed in TLR2 and TLR1/6-like (Table 2). The absence of non-synonymous mutations may be the result of selective pressures [69]. Purifying selection appears to be acting on TLR2 and TLR4 (Table 4) and it is tempting to speculate that mutations in the pathogen-binding region of extracellular domain in these genes may adversely affect fitness. In future it will be interesting to investigate whether there is an association between these genes and response to disease. Polymorphisms at human TLR2 and TLR4 have been found to be associated with the inflammation and increased disease susceptibility. For instance, SNPs Arg753Gln and Arg677Trp at TLR2 contribute to the course of sepsis [70] and D299G and T399I at TLR4 are associated with infections caused by *Leginoella pneumophila* [71].

**Table 4:** Selection test at nine koala TLRs.

	**Substitution rate**			**Z test of neutral selection (d**_N_ **≠d**_S_ **)**		**Z test of purifying selection (d**_N_ **<d**_S_ **)**	
**Gene**	**d**_N_	**d**_S_	**d**_N_ **-d**_S_	**Statistic**	**P**	**Statistic**	**P**
TLR2	0	0.003	−0.003	−1.823	0.071	1.801	0.037*
TLR3	0	0.004	−0.004	−1.565	0.12	1.552	0.062
TLR4	0.001	0.004	−0.003	−1.634	0.105	1.803	0.036*
TLR5	0.001	0.002	−0.001	−0.938	0.35	0.926	0.178
TLR1/6like	0	0.002	−0.002	−1.038	0.301	1.031	0.152
TLR7	0.001	0.002	−0.001	−1.362	0.176	1.371	0.086
TLR8	0	0.001	−0.001	−0.969	0.334	0.971	0.167
TLR9	0.001	0.002	−0.002	−1.574	0.118	1.571	0.059
TLR13	0.001	0	0.001	1.059	0.292	-1.078	1.000

*Significant purifying selection p ≤0.05

Given the locations that these koalas were sampled from, it is likely that all of the koalas tested in this study were KoRV positive based on previous observations that koalas in north-eastern NSW were 100% KoRV-positive [72]. Nothing is known about the chlamydial infection status of the animals sampled in this preliminary study, limiting any further commentary on the association between TLR variants and koala disease. While this is an unfortunate limitation, future studies utilising the TLR molecular markers described here alongside the general availability of tissue samples from *Chlamydia*-free, natural *Chlamydia*-infected but clinically healthy koalas and koalas that have developed severe *Chlamydia*-related ocular or reproductive tract pathology means that koala researchers will now be in a strong position to investigate whether TLRs have an impact on koala chlamydial disease pathogenesis. To this end, work in naturally infected women has already provided the first suggestions that TLR sequence variation may be associated with increased risk of infection and inflammatory disease [29], but such studies have been generally limited by sample size. With the recent availability of genetic resources for the koala [73], expanded koala studies such as those proposed may provide new insights into host genetic susceptibility more generally while also providing stakeholders with important management information that can be used to deploy conservation tools such as a prototype koala chlamydial vaccine [74].

## Supporting Information

S1 Fig Mitochondrial Haplotypes. Haplotype network showing stepwise sequence divergence, for koala samples from NSW used in this study. Each step depicts a nucleotide difference between haplotypes. H12, 18 and H5 previously reported by [5], and Q1 previously reported by [6]. 1, 2, 3, 6, 8 all novel haplotypes previously unreported.
(TIF)

S2 Fig Koala TLR2 Nucleotide and Amino acid sequences. Boxes and arrows means the locations of primers (→: forward primer, ←: reverse primer). Vertical lines show the boundaries of Leucine-Rich Repeats, transmembrane and cytoplasmic region.
(TIF)

S3 Fig Koala TLR3 Nucleotide and Amino acid sequences. (TIF)

S4 Fig Koala TLR4 Nucleotide and Amino acid sequences. (TIF)

S5 Fig Koala TLR5 Nucleotide and Amino acid sequences. (TIF)

S6 Fig Koala TLR1/6like Nucleotide and Amino acid sequences. (TIF)

S7 Fig Koala TLR7 Nucleotide and Amino acid sequences. (TIF)

S8 Fig Koala TLR8 Nucleotide and Amino acid sequences. (TIF)

S9 Fig Koala TLR9 Nucleotide and Amino acid sequences. (TIF)

S10 Fig Koala TLR10 Nucleotide and Amino acid sequences. (TIF)

S11 Fig Koala TLR13 Nucleotide and Amino acid sequences. (TIF)

S12 Fig Amino acid alignment at TLR2. Amino acid alignment of coding sequences of TLR2 in koala, Tasmanian devil, gray short-tailed opossum, house mouse, cattle and human. Dashes in the sequences represent gaps. Dots represent conservation of amino acids with the koala sequence. The ruler has been adjusted according to the koala TLR sequence. The LRR motifs are in green and are marked above with the consensus pattern: LxxLxLxxNxL according to human TLRs (“L” is Leu, Ile, Val, or Phe. “N” is Asn, Thr, Ser, or Cys. “x” represent residue.) [54]. The positions of the β-strand are shown as arrows above the sequences [58]. The stars above the residues indicate predicted pathogen binding positions in human [58].
(TIF)

S13 Fig Amino acid alignment at TLR3. The stars above the residues are predicted pathogen binding positions in human TLR3 [53]. The predicted pathogen binding positions are in box according to human TLR3 [54]. The “^” above the residues indicate sites of non-synonymous substitutions.
(TIF)

S14 Fig Amino acid alignment at TLR4. The predicted pathogen binding positions are in box according to human TLR4 [55].
(TIF)

S15 Fig Amino acid alignment at TLR5. The predicted pathogen binding positions are in box according to human TLR5 [56].
(TIF)

S16 Fig Amino acid alignment at TLR1/6like. The predicted pathogen binding positions are in box according to human TLR6 [25].
(TIF)

S17 Fig Amino acid alignment at TLR7. The predicted pathogen binding positions are in box according to human TLR7 [25].
(TIF)

S18 Fig Amino acid alignment at TLR8. The predicted pathogen binding positions are in box according to human TLR8 [25].
(TIF)

S19 Fig Amino acid alignment at TLR9. The predicted pathogen binding positions are in box according to human TLR9 [25].
(TIF)

S20 Fig Amino acid alignment at TLR10. The predicted pathogen binding positions are in box according to human TLR10 [25].
(TIF)

S21 Fig Amino acid alignment at TLR13. (TIF)

S1 Table Accession numbers of Toll-like receptor sequences used in this study. Amino acid sequence accession numbers were all obtained from GenBank, except *Xenopus tropicalis* TLR9 that was from Ensemble.
(XLS)

S2 Table Australian Museum registration numbers of investigated koalas. (XLS)

S3 Table GenBank accession number of koala TLRs. (XLS)

S4 Table Genotype of TLRs and mitochondrial DNA. (XLS)
