# Supplementary figures and images for: SNP Marker Discovery in Koala TLR Genes
Source: PLoS One. 2015 Mar 23;10(3):e0121068. doi: 10.1371/journal.pone.0121068 (PMC4370664; doi:10.1371/journal.pone.0121068)

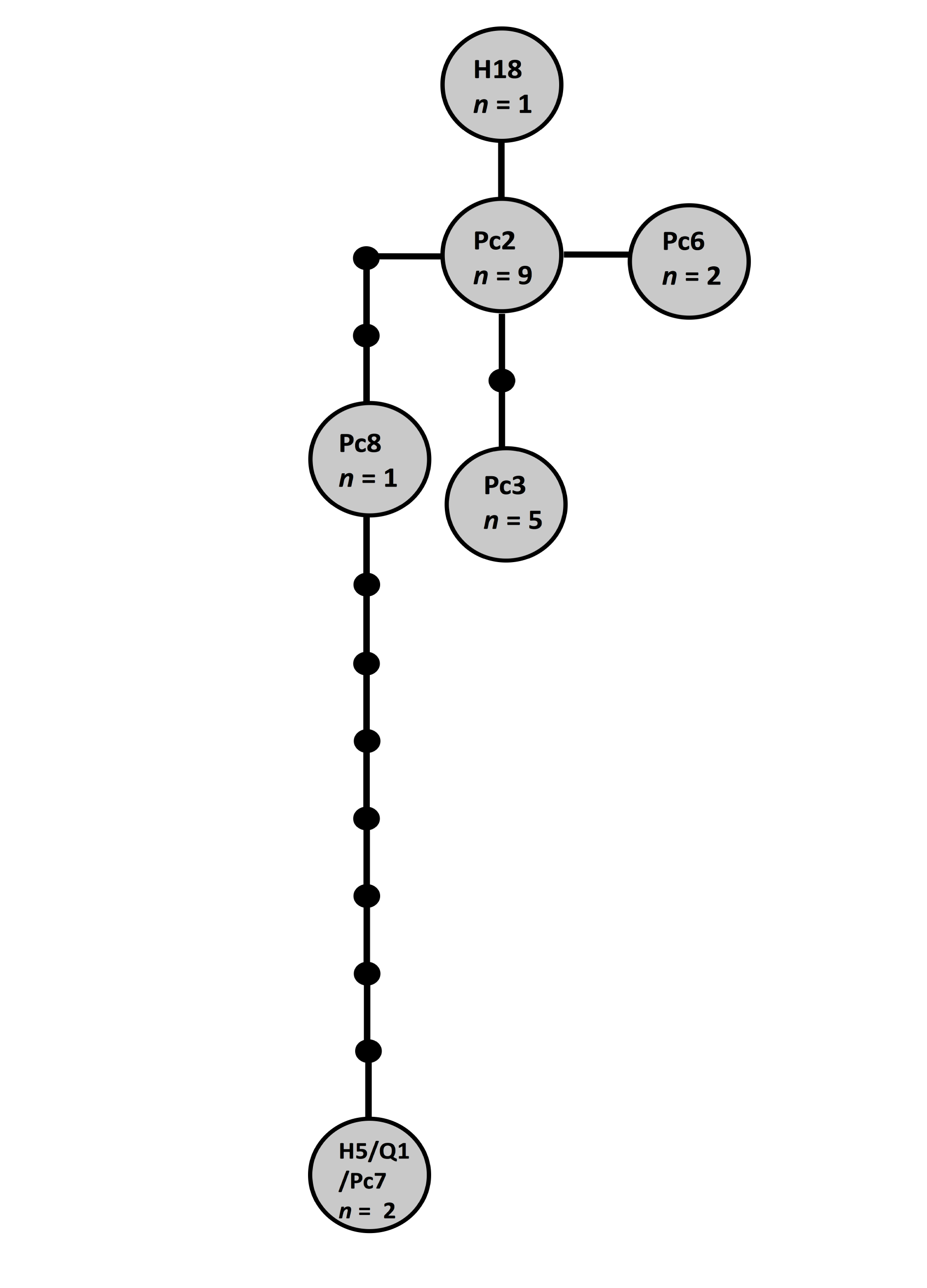

Supplement: S1 Fig — Haplotype network showing stepwise sequence divergence, for koala samples from NSW used in this study. Each step depicts a nucleotide difference between haplotypes. H12, 18 and H5 previously reported by [5], and Q1 previously reported by [6]. 1, 2, 3, 6, 8 all novel haplotypes previously unreported. (TIF) [file pone.0121068.s001.tif]

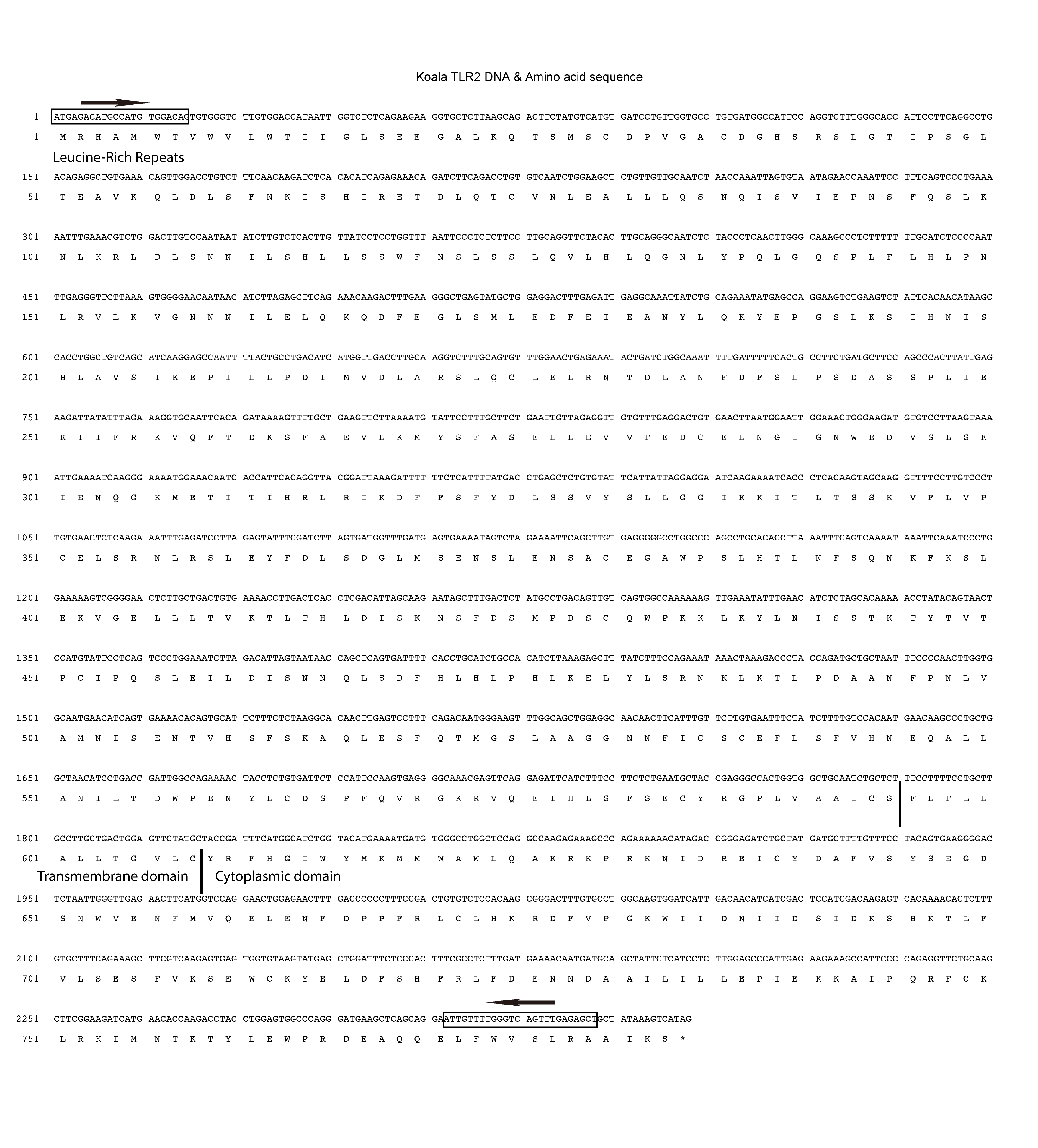

Supplement: S2 Fig — Boxes and arrows means the locations of primers (→: forward primer, ←: reverse primer). Vertical lines show the boundaries of Leucine-Rich Repeats, transmembrane and cytoplasmic region. (TIF) [file pone.0121068.s002.tif]

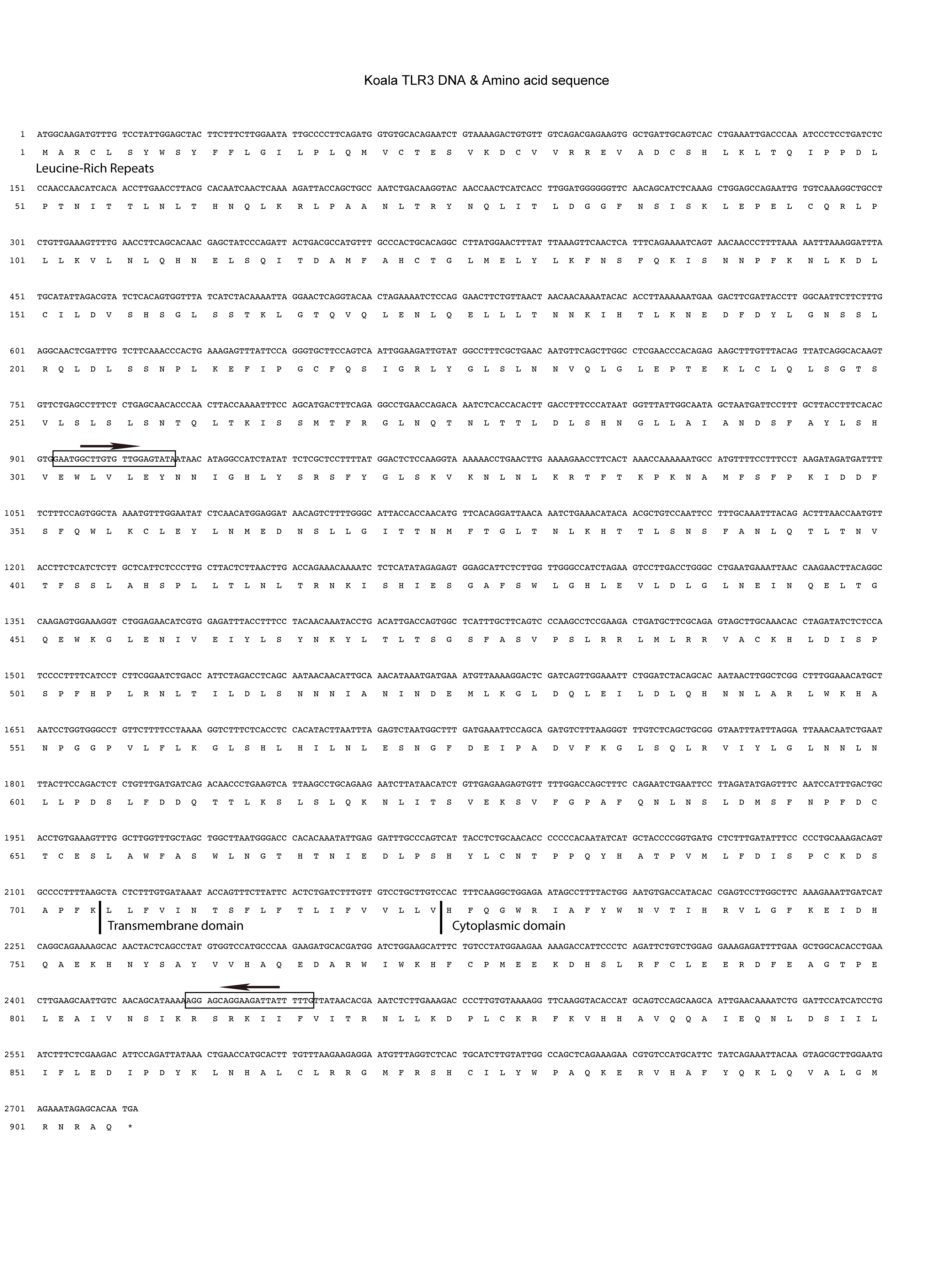

Supplement: S3 Fig — (TIF) [file pone.0121068.s003.tif]

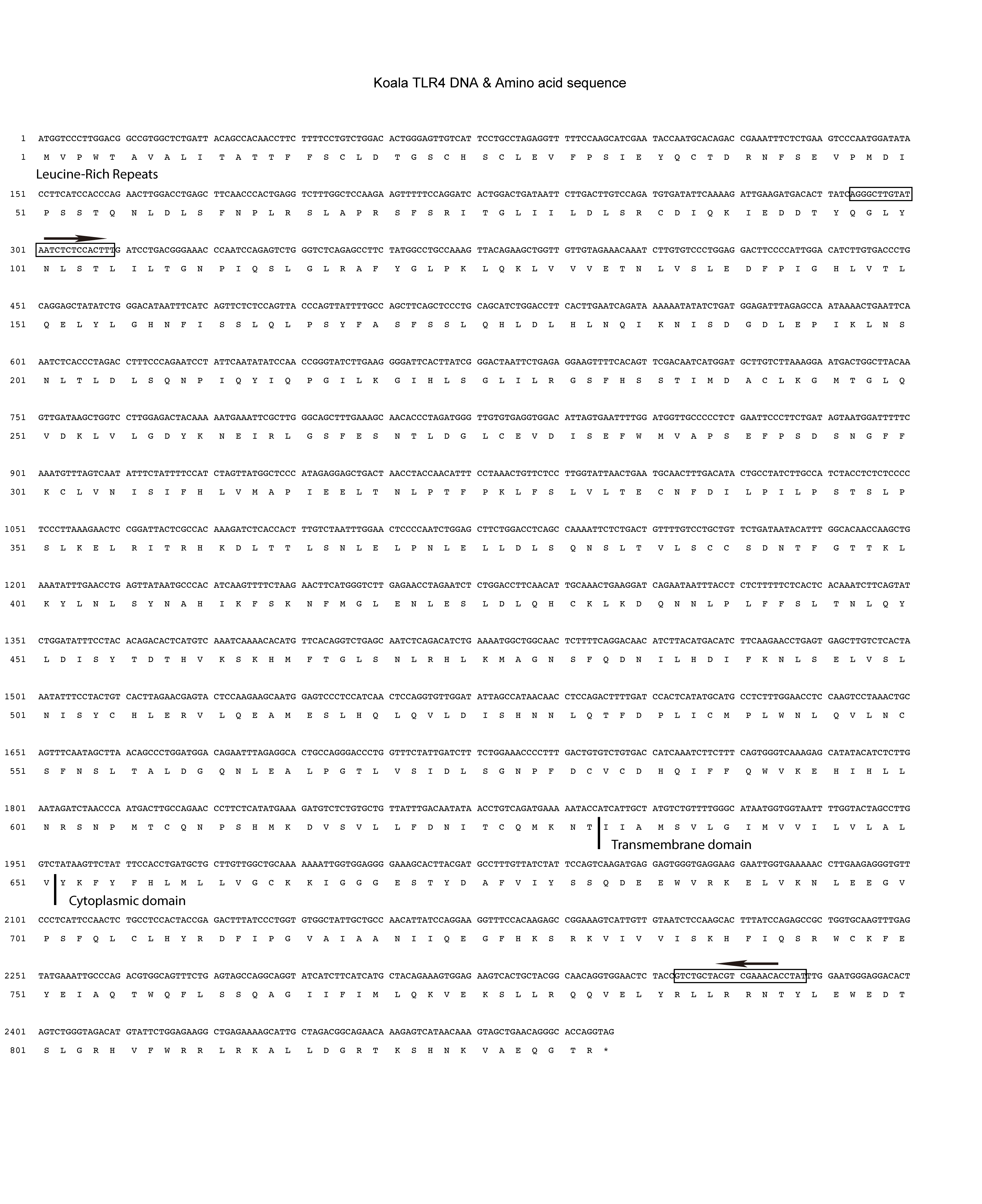

Supplement: S4 Fig — (TIF) [file pone.0121068.s004.tif]

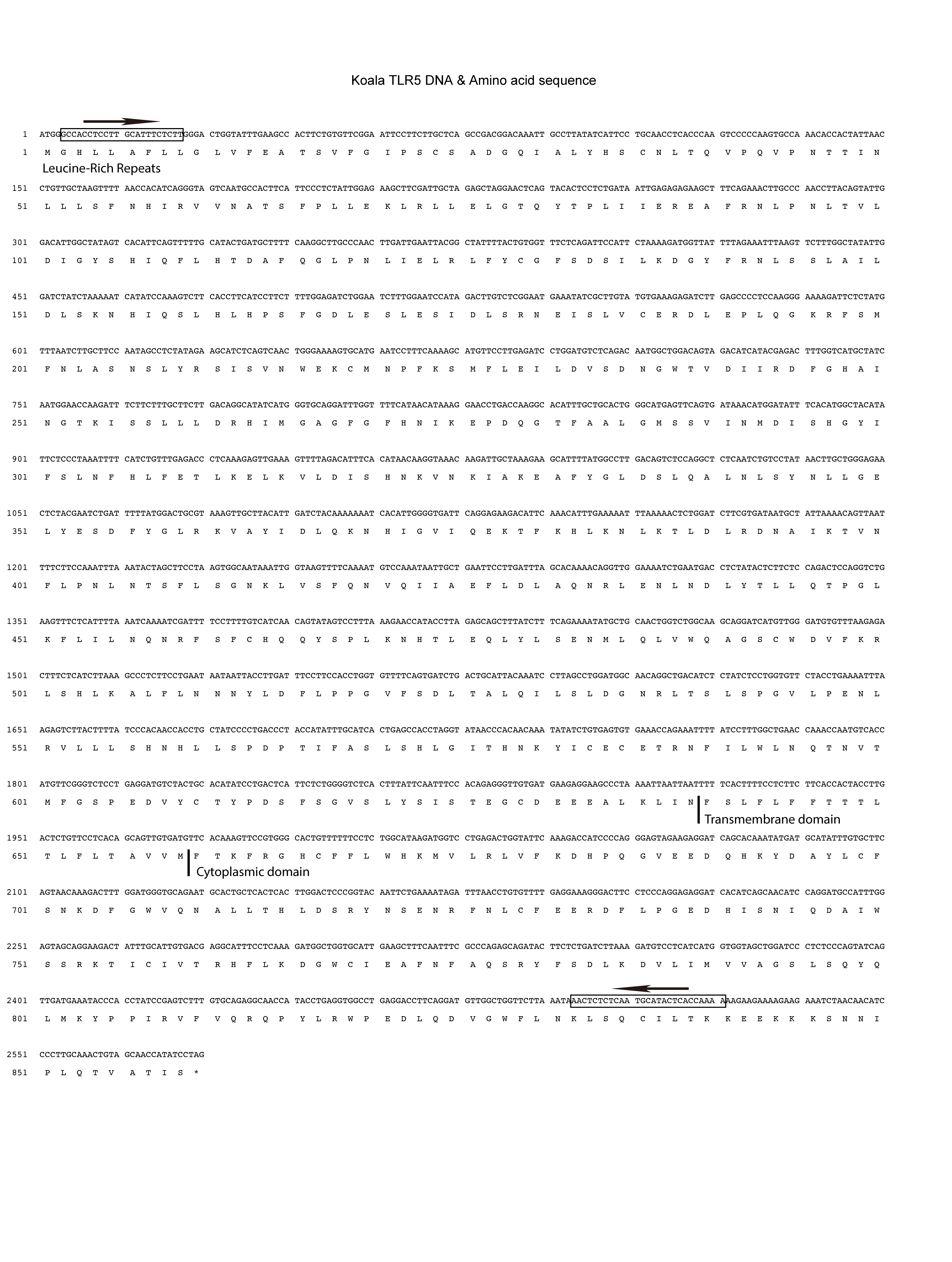

Supplement: S5 Fig — (TIF) [file pone.0121068.s005.tif]

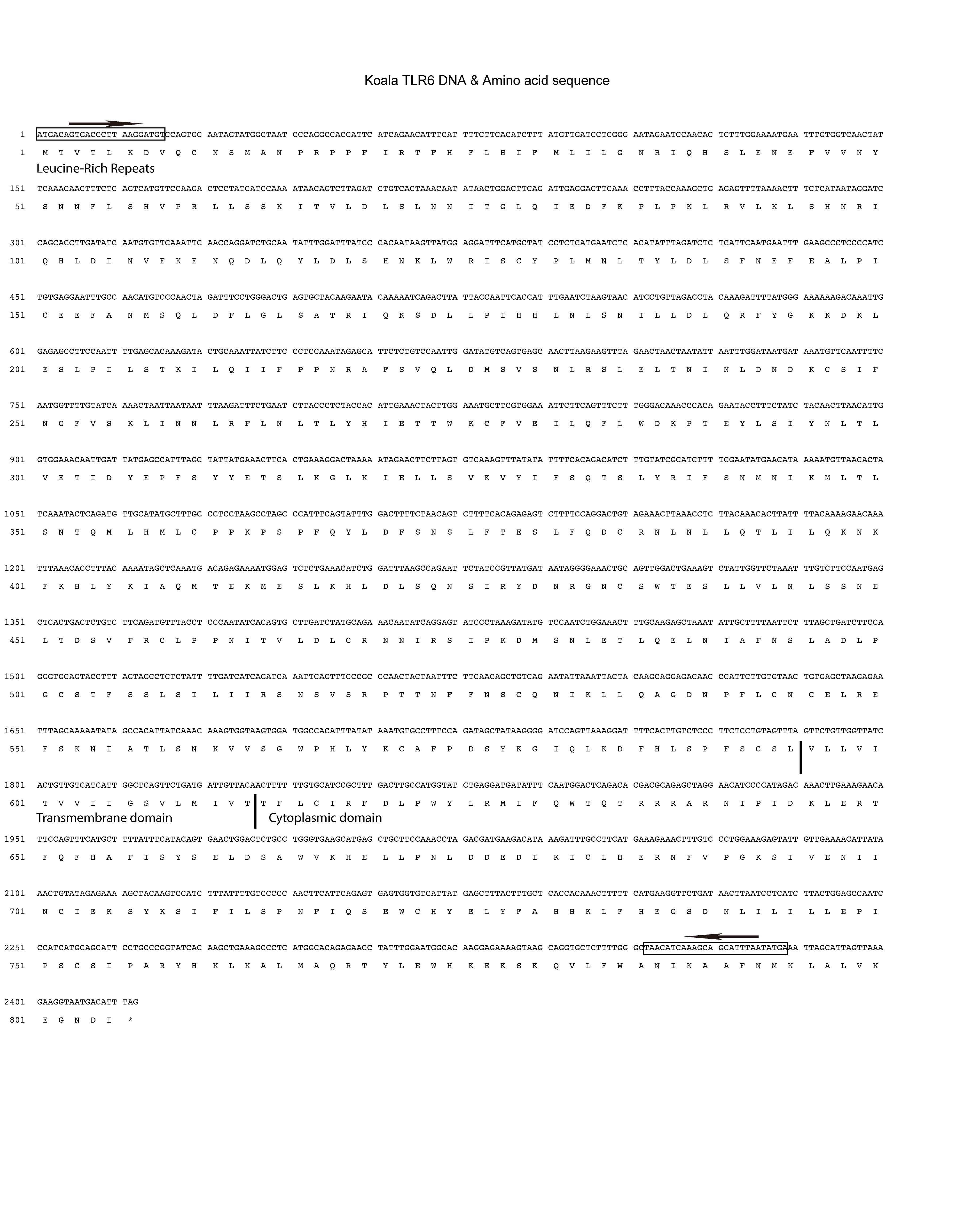

Supplement: S6 Fig — (TIF) [file pone.0121068.s006.tif]

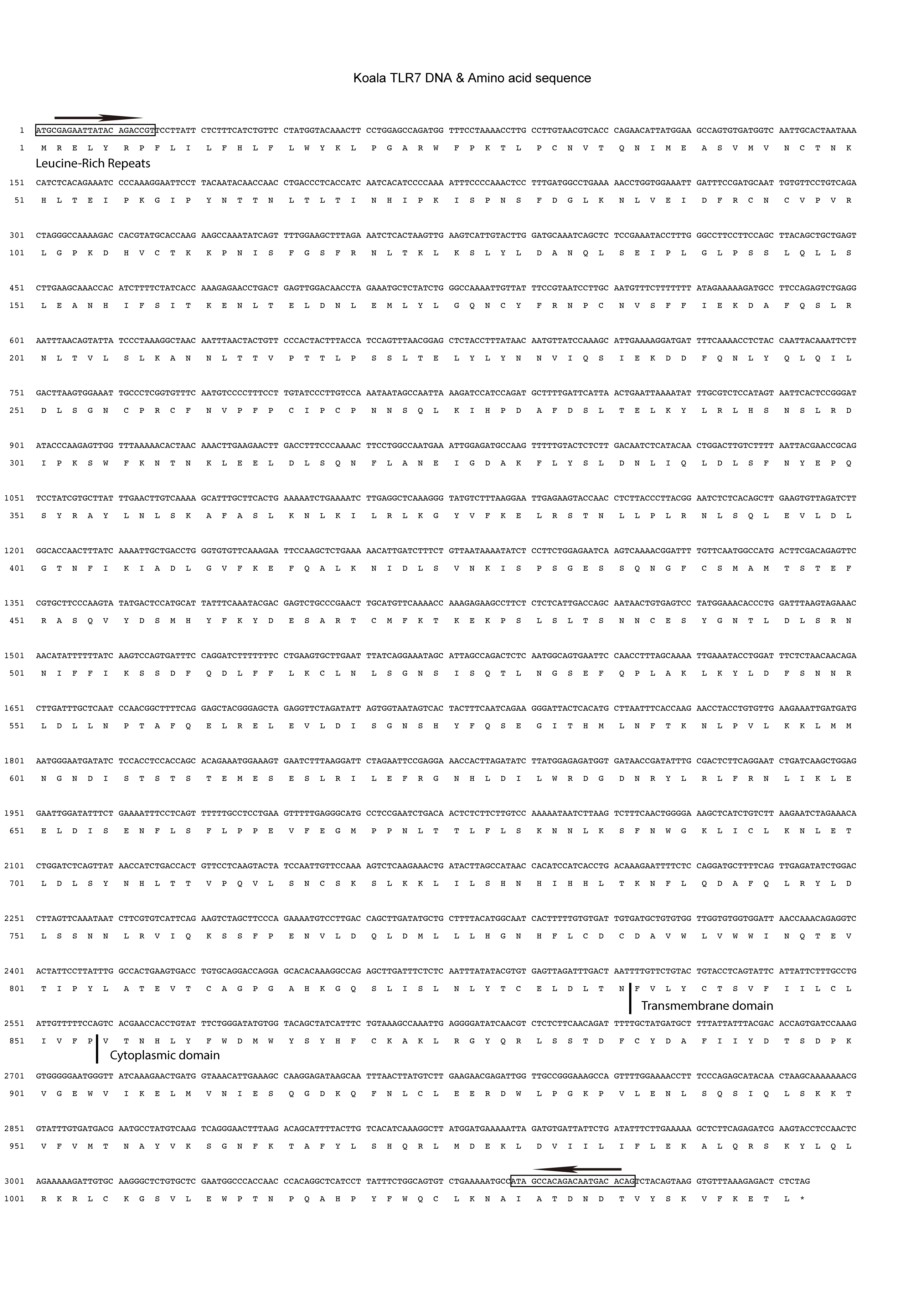

Supplement: S7 Fig — (TIF) [file pone.0121068.s007.tif]

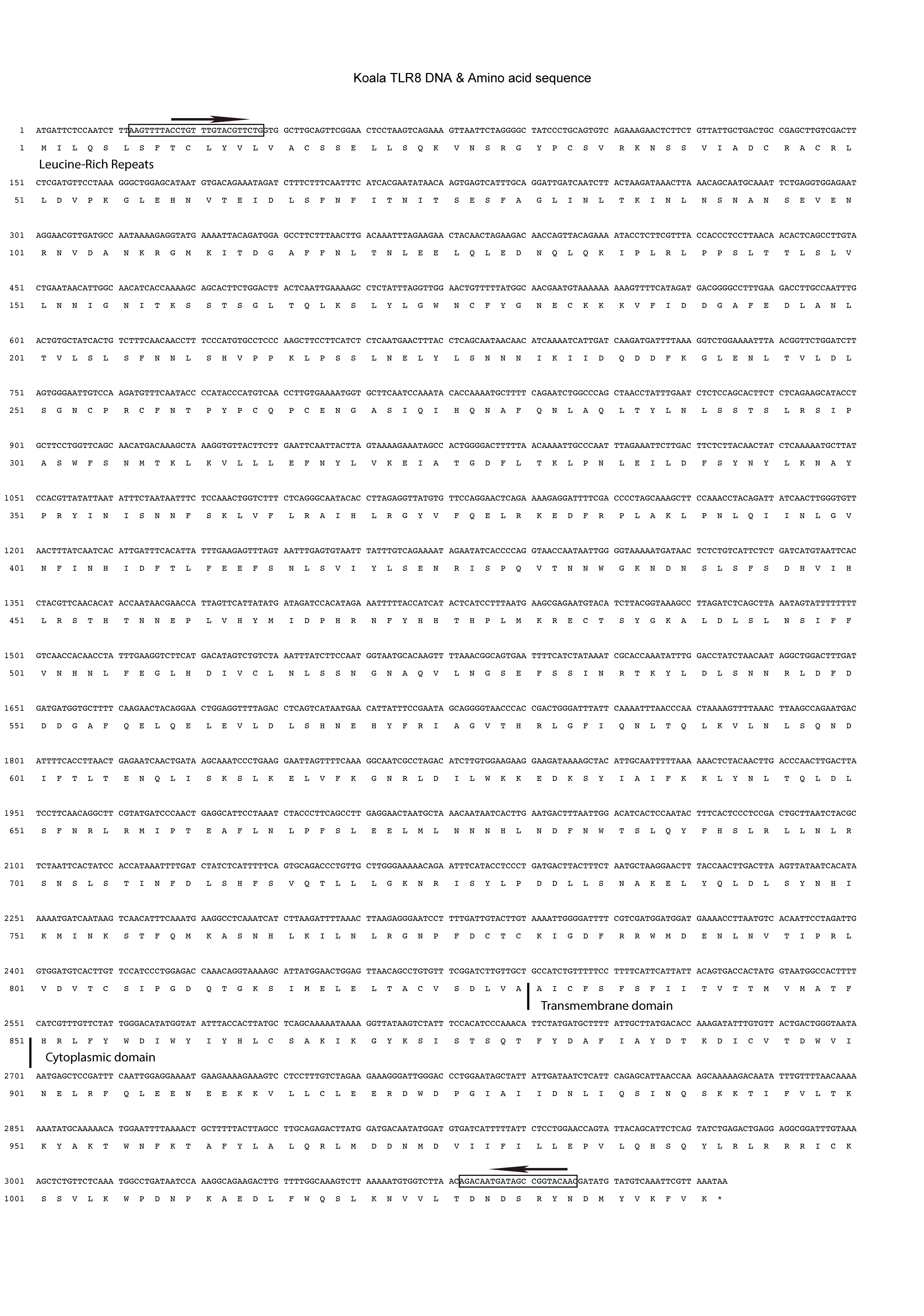

Supplement: S8 Fig — (TIF) [file pone.0121068.s008.tif]

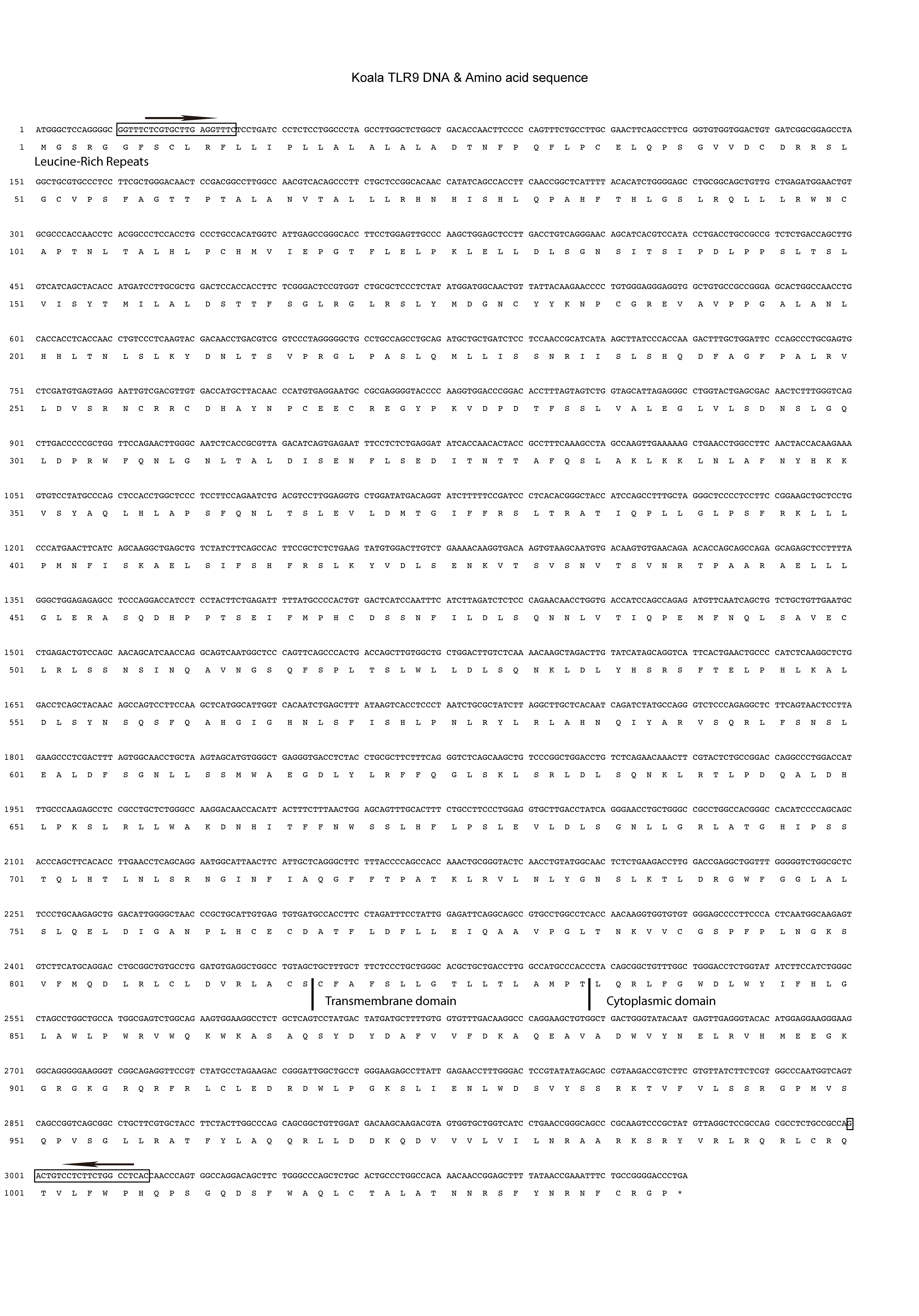

Supplement: S9 Fig — (TIF) [file pone.0121068.s009.tif]

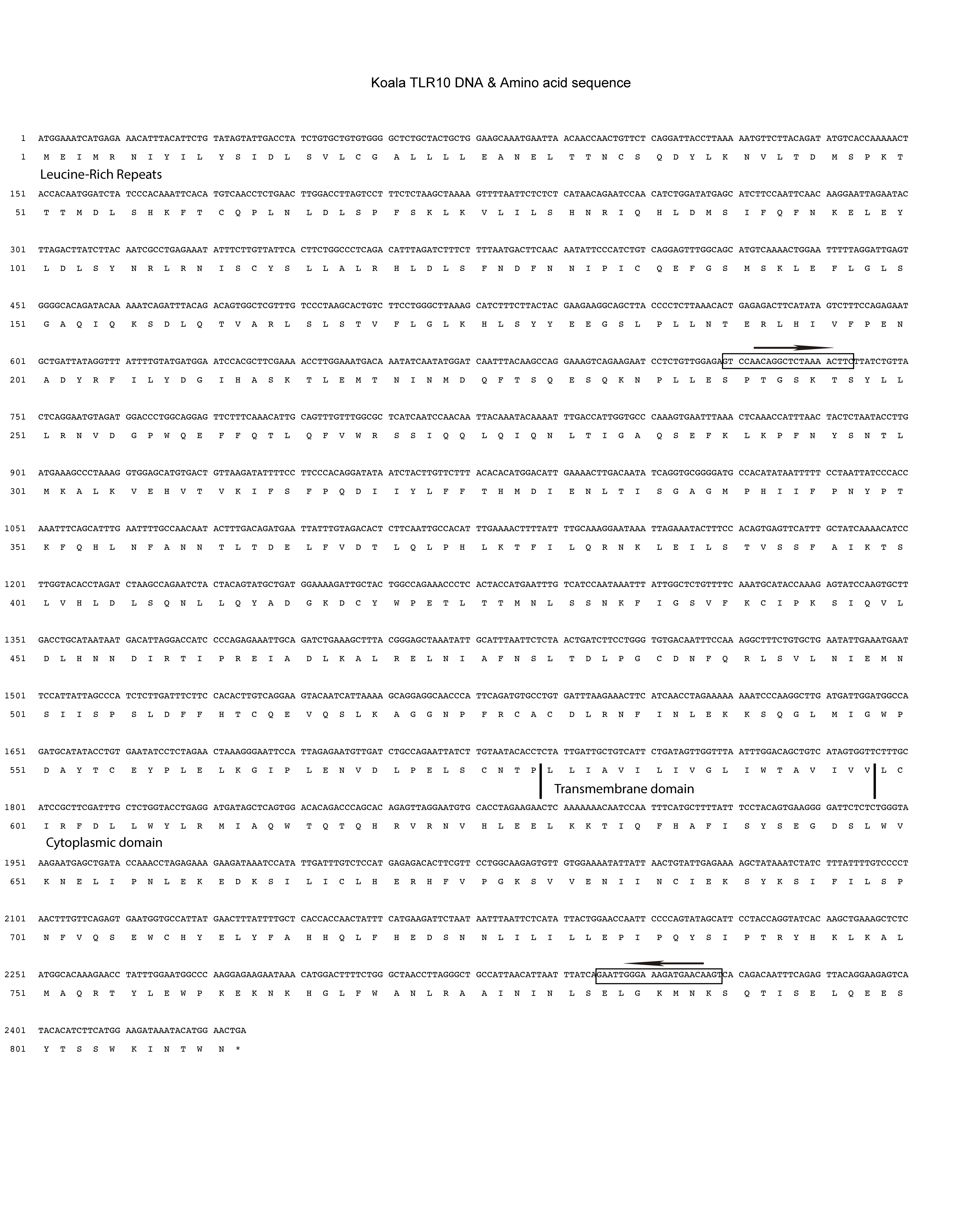

Supplement: S10 Fig — (TIF) [file pone.0121068.s010.tif]

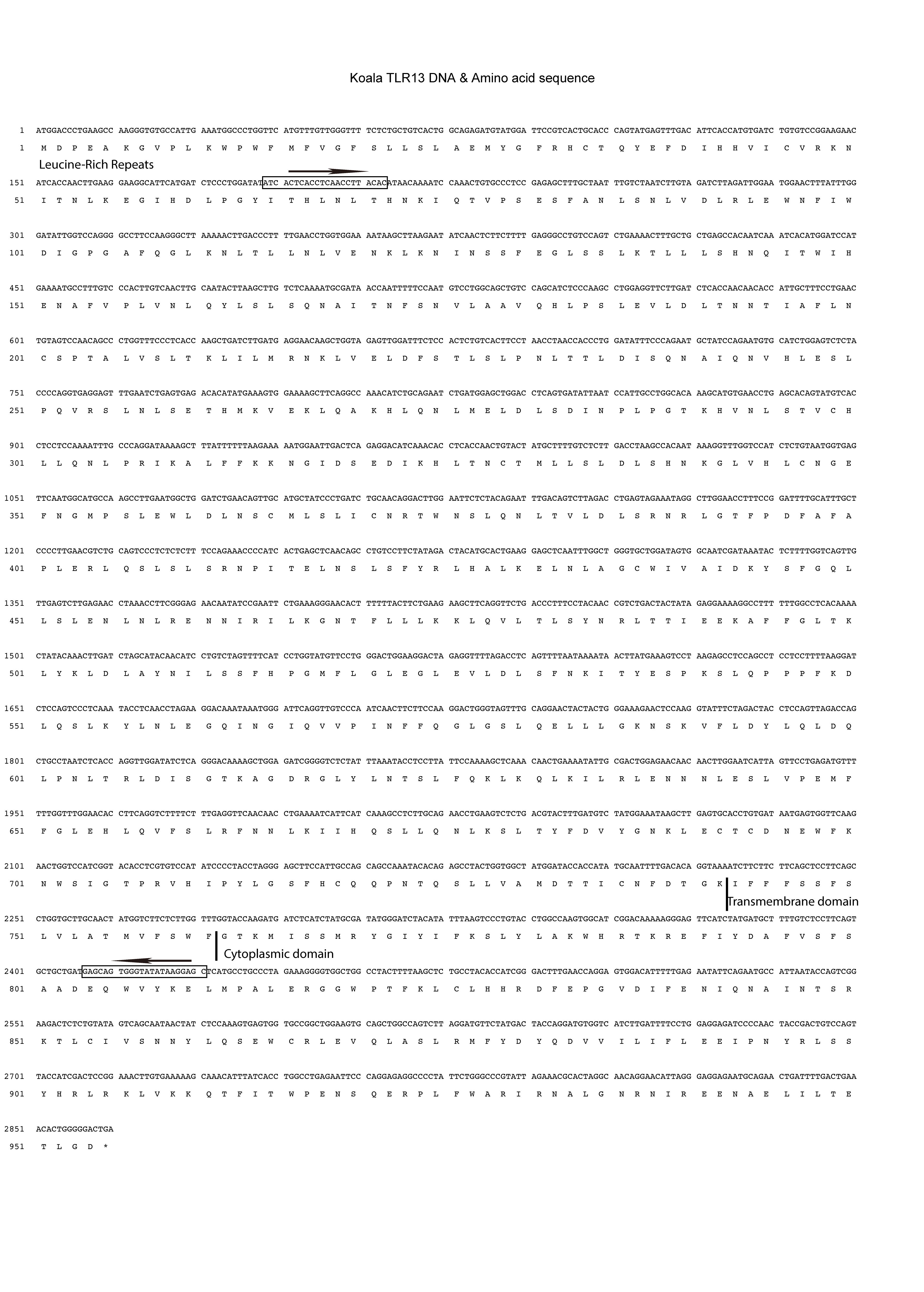

Supplement: S11 Fig — (TIF) [file pone.0121068.s011.tif]

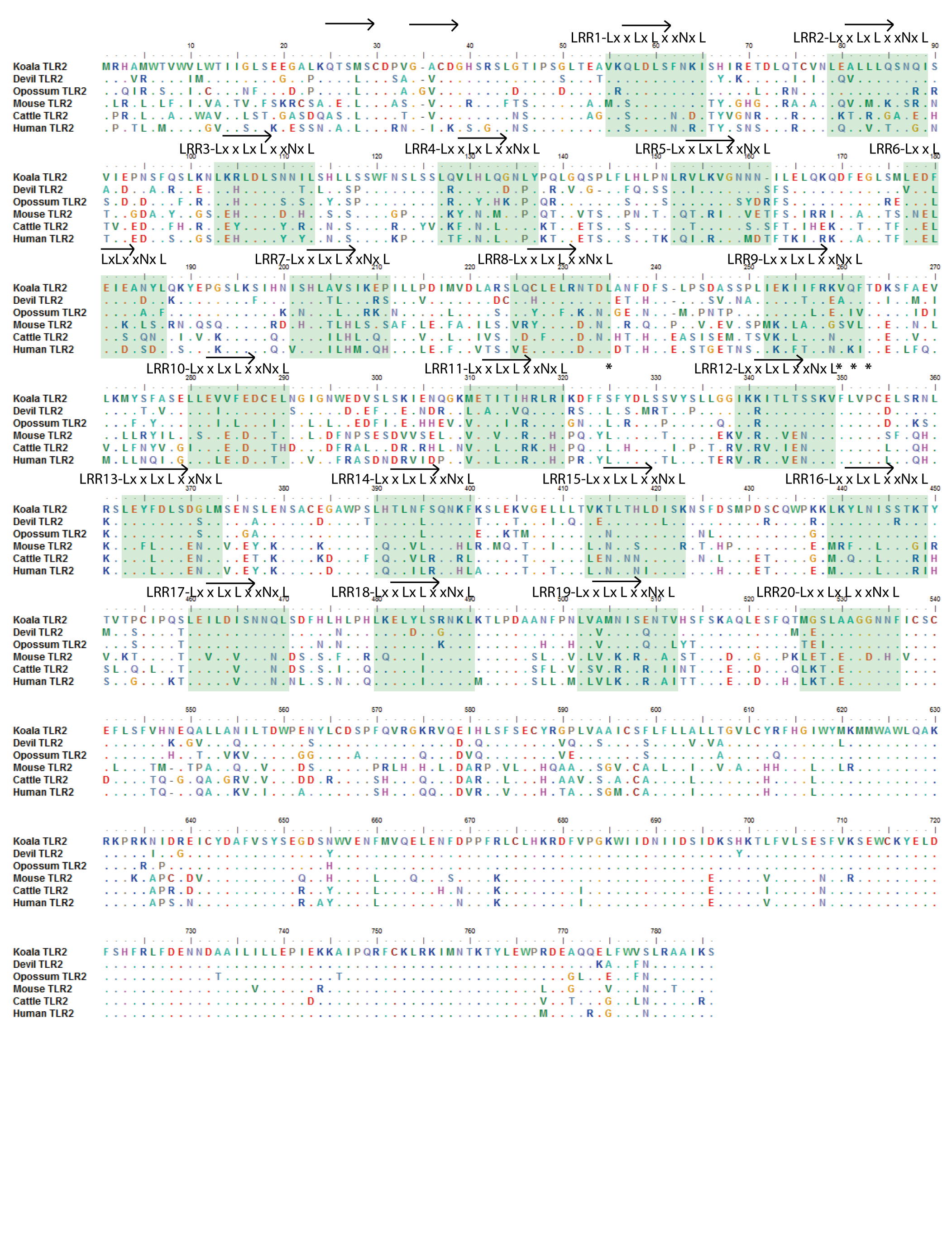

Supplement: S12 Fig — Amino acid alignment of coding sequences of TLR2 in koala, Tasmanian devil, gray short-tailed opossum, house mouse, cattle and human. Dashes in the sequences represent gaps. Dots represent conservation of amino acids with the koala sequence. The ruler has been adjusted according to the koala TLR sequence. The LRR motifs are in green and are marked above with the consensus pattern: LxxLxLxxNxL according to human TLRs (“L” is Leu, Ile, Val, or Phe. “N” is Asn, Thr, Ser, or Cys. “x” represent residue.) [54]. The positions of the β-strand are shown as arrows above the sequences [58]. The stars above the residues indicate predicted pathogen binding positions in human [58]. (TIF) [file pone.0121068.s012.tif]

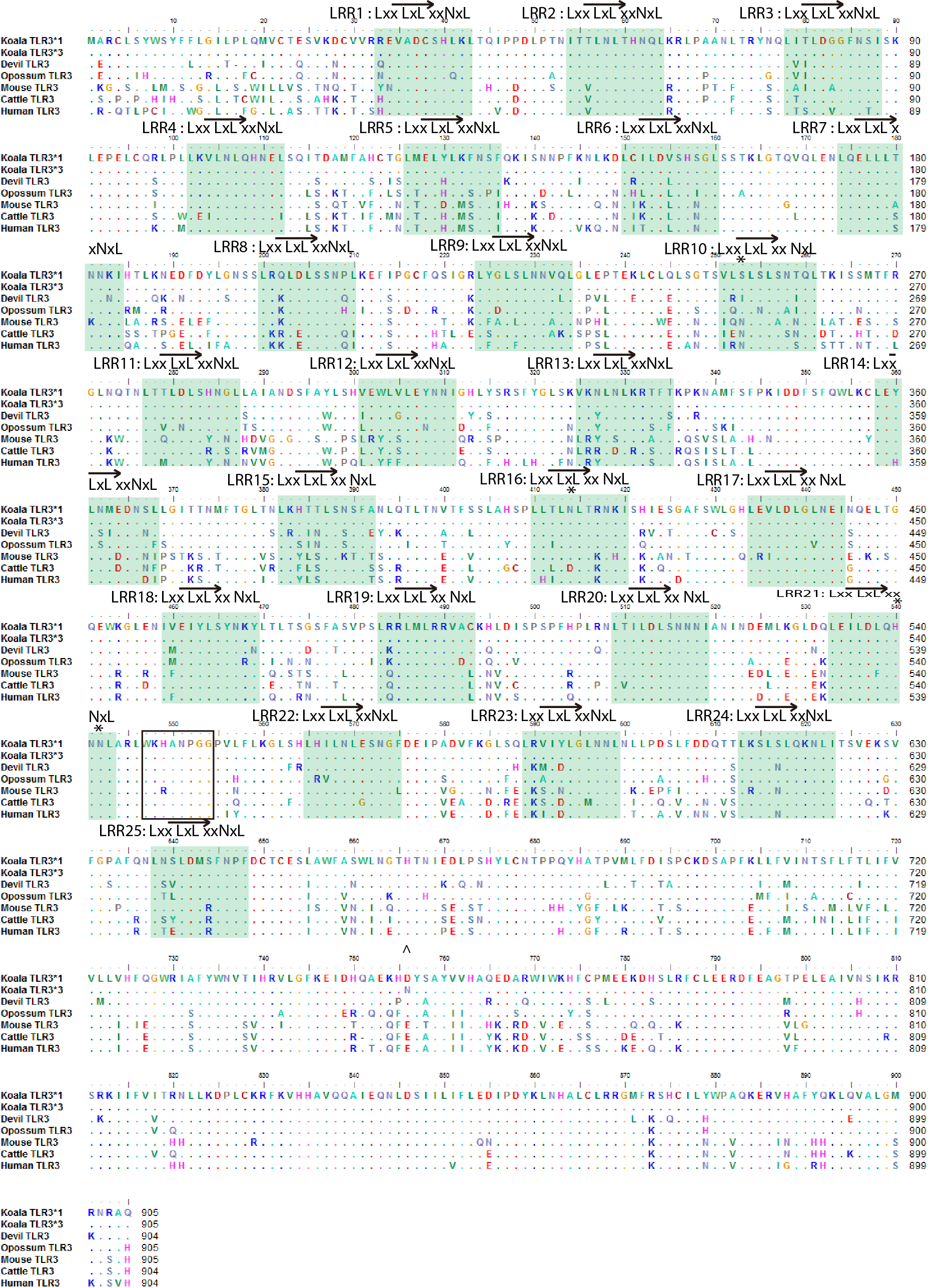

Supplement: S13 Fig — The stars above the residues are predicted pathogen binding positions in human TLR3 [53]. The predicted pathogen binding positions are in box according to human TLR3 [54]. The “^” above the residues indicate sites of non-synonymous substitutions. (TIF) [file pone.0121068.s013.tif]

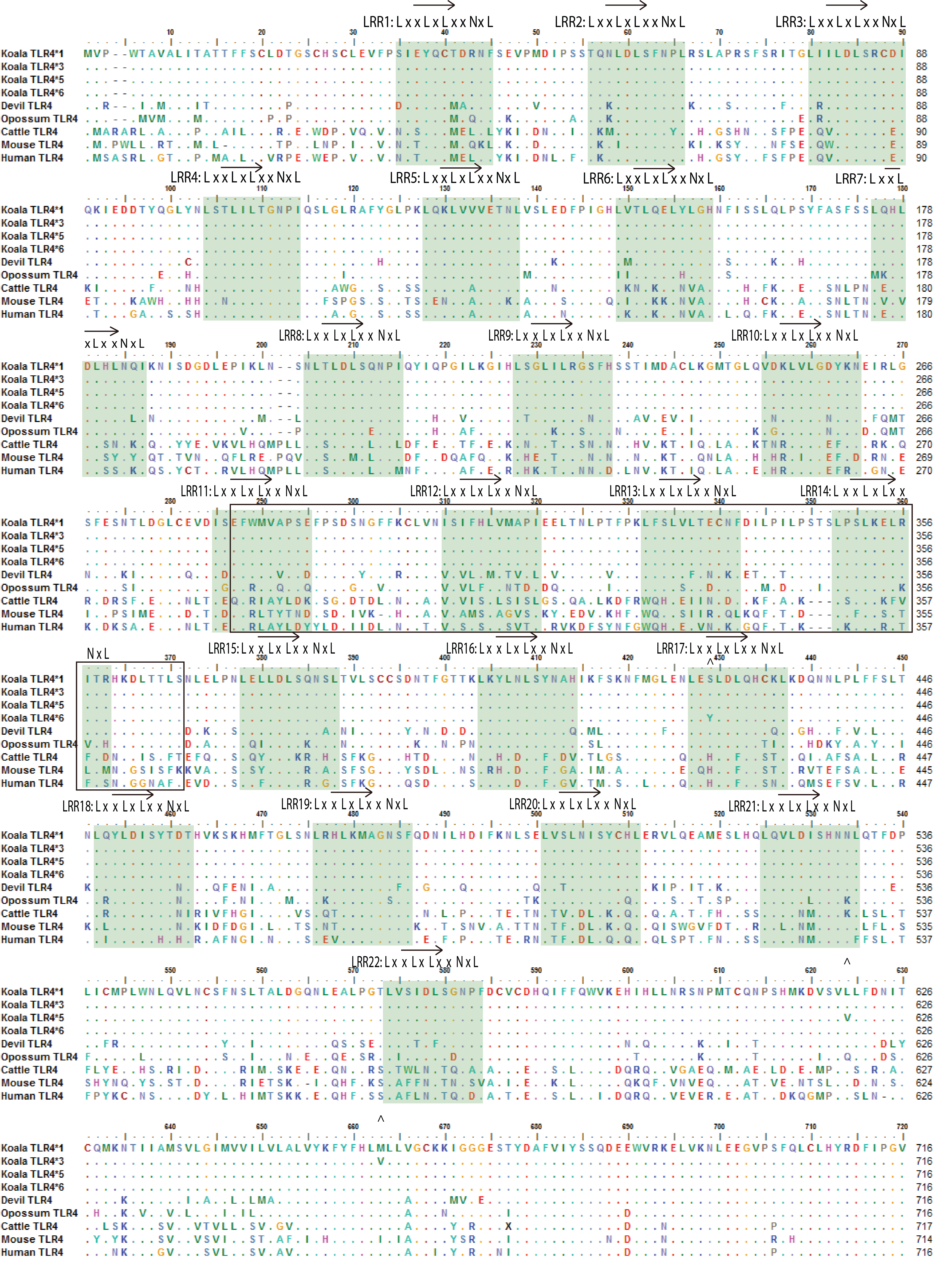

Supplement: S14 Fig — The predicted pathogen binding positions are in box according to human TLR4 [55]. (TIF) [file pone.0121068.s014.tif]

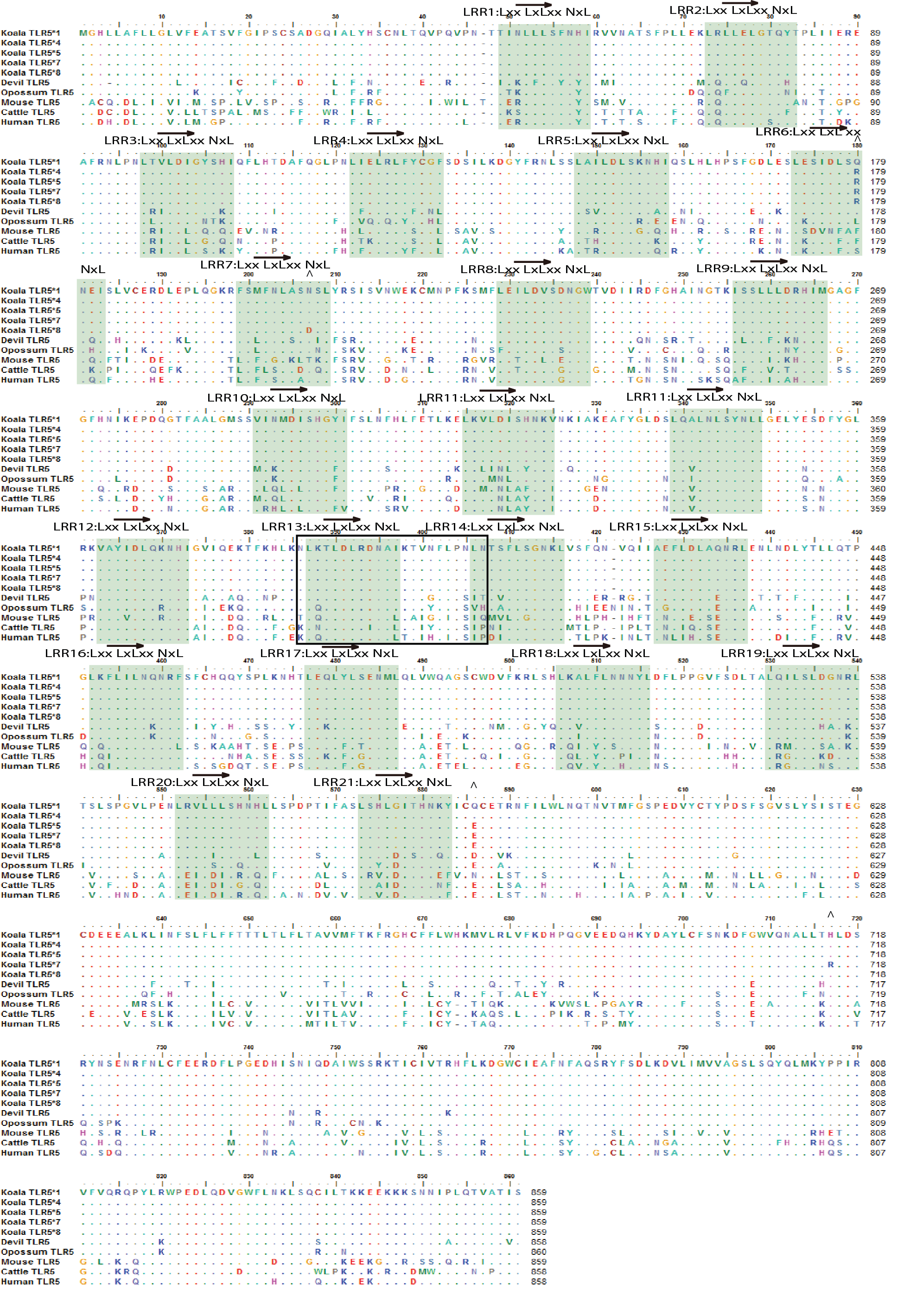

Supplement: S15 Fig — The predicted pathogen binding positions are in box according to human TLR5 [56]. (TIF) [file pone.0121068.s015.tif]

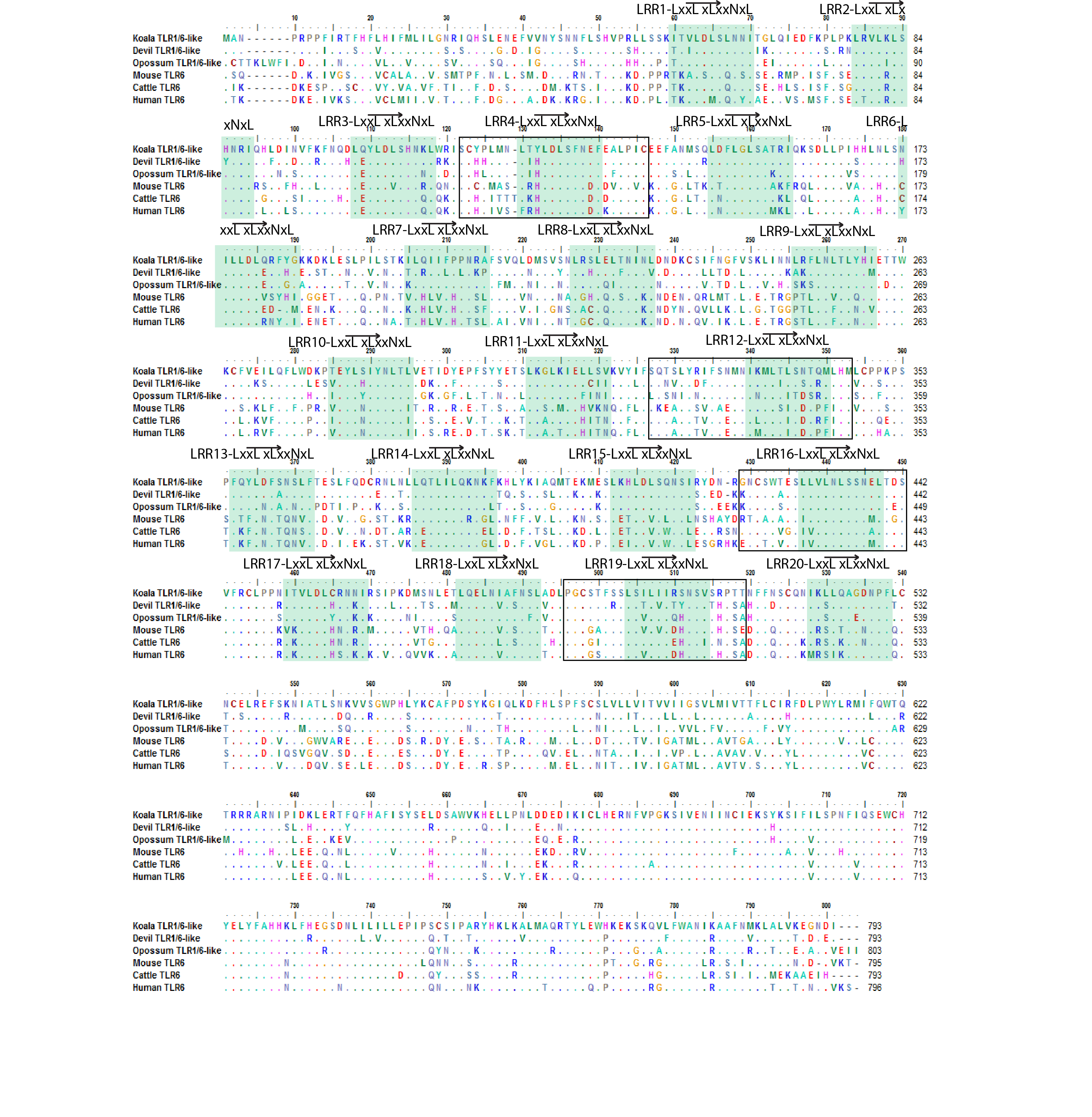

Supplement: S16 Fig — The predicted pathogen binding positions are in box according to human TLR6 [25]. (TIF) [file pone.0121068.s016.tif]

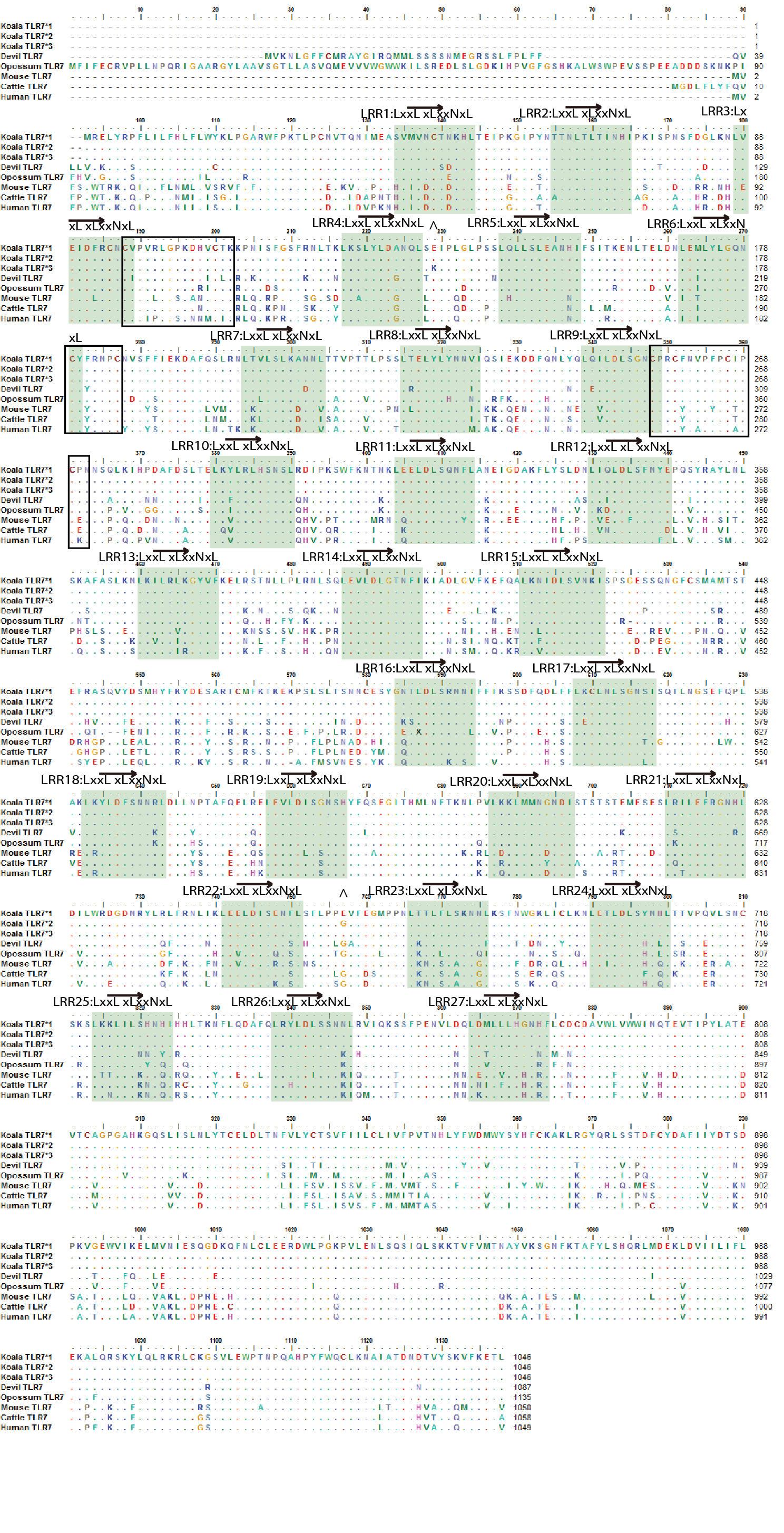

Supplement: S17 Fig — The predicted pathogen binding positions are in box according to human TLR7 [25]. (TIF) [file pone.0121068.s017.tif]

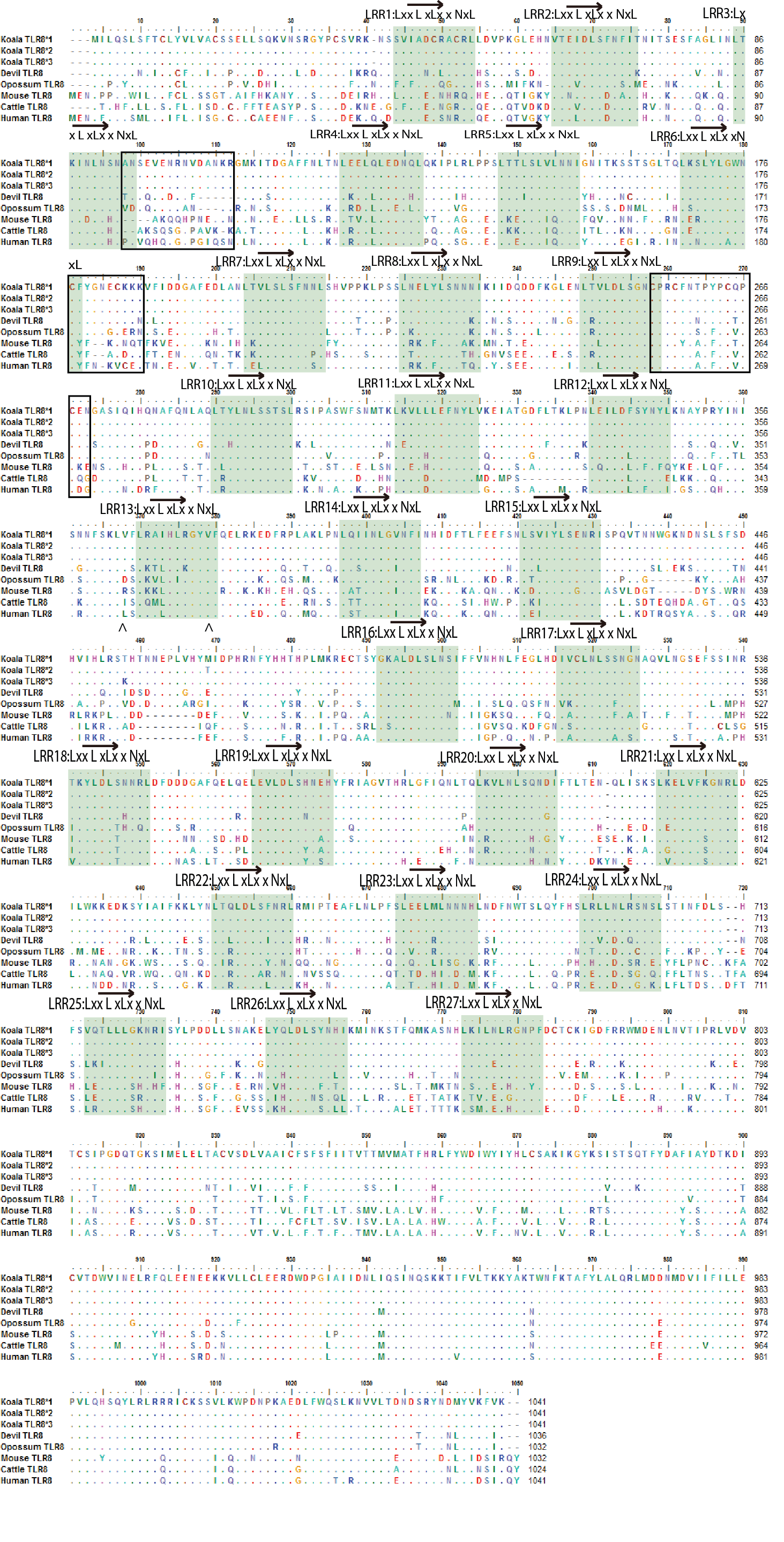

Supplement: S18 Fig — The predicted pathogen binding positions are in box according to human TLR8 [25]. (TIF) [file pone.0121068.s018.tif]

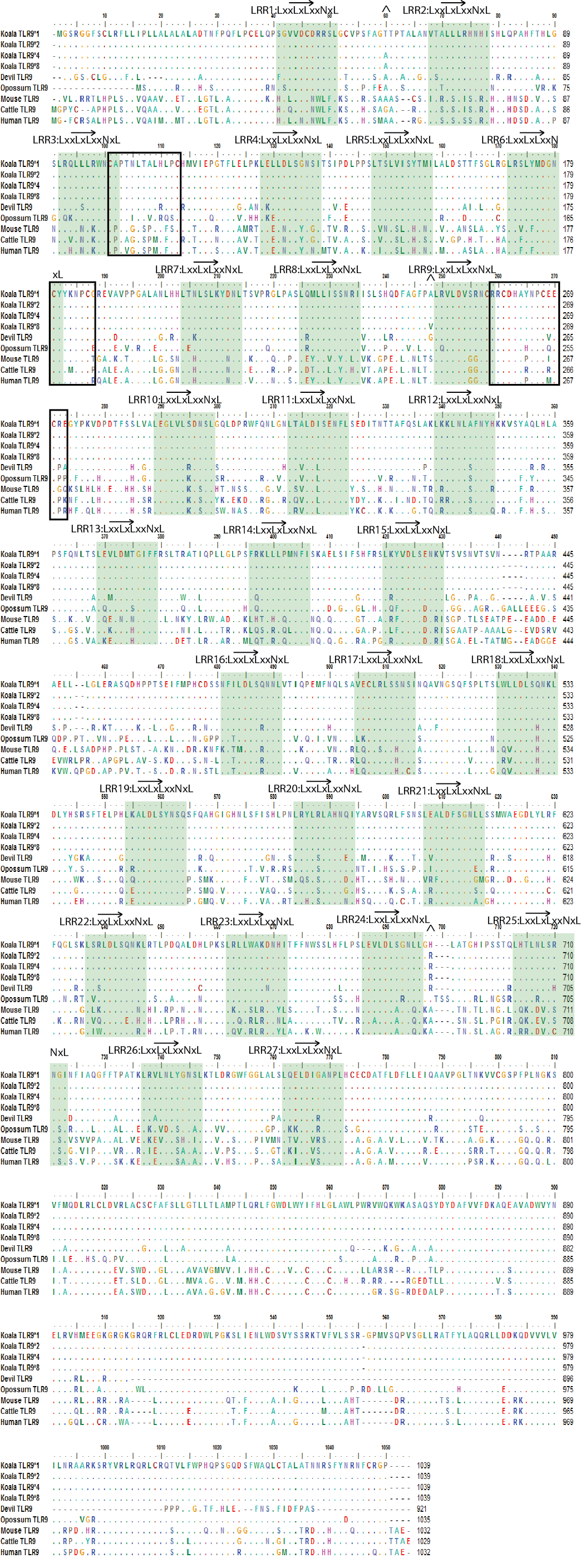

Supplement: S19 Fig — The predicted pathogen binding positions are in box according to human TLR9 [25]. (TIF) [file pone.0121068.s019.tif]

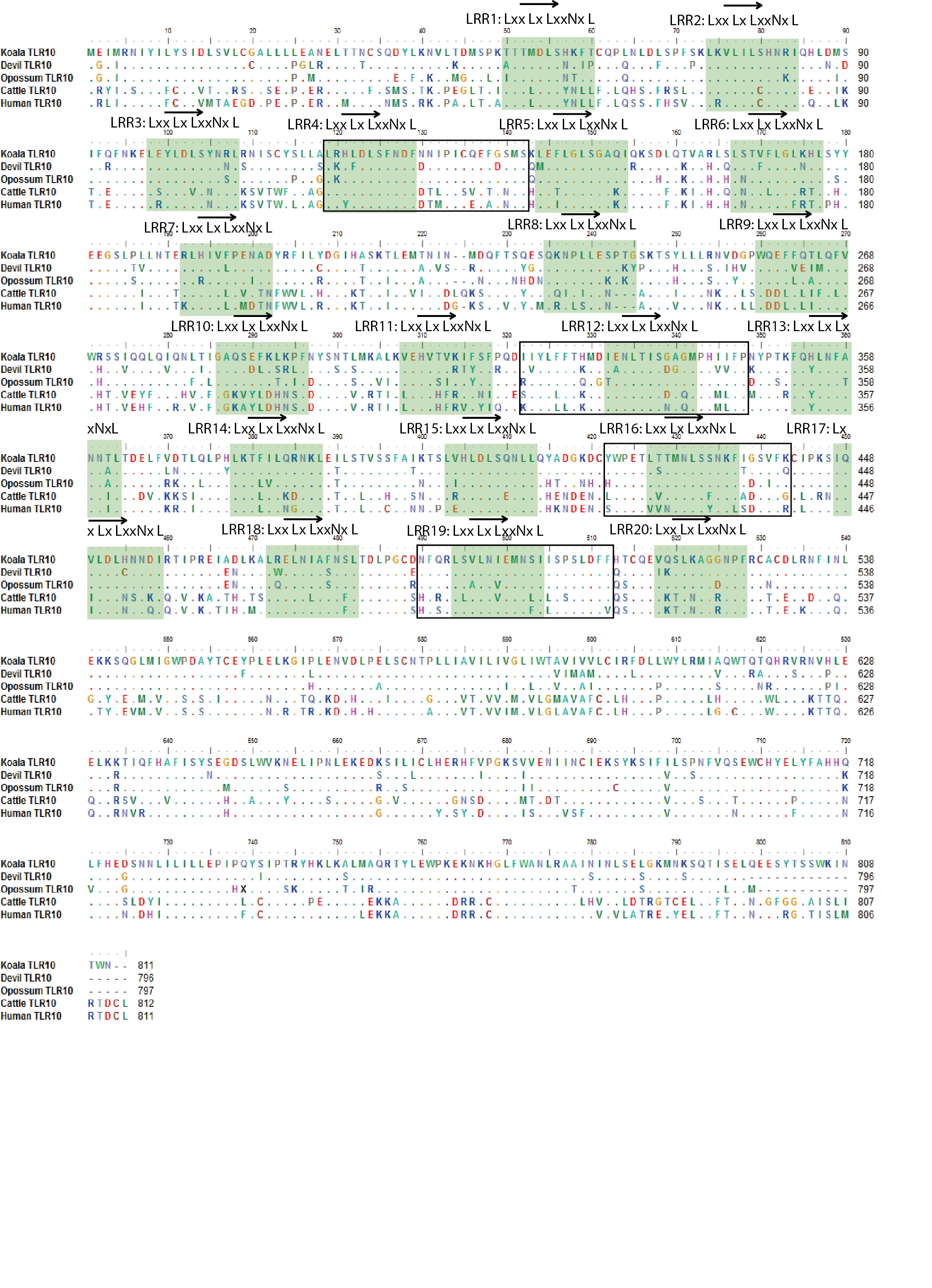

Supplement: S20 Fig — The predicted pathogen binding positions are in box according to human TLR10 [25]. (TIF) [file pone.0121068.s020.tif]

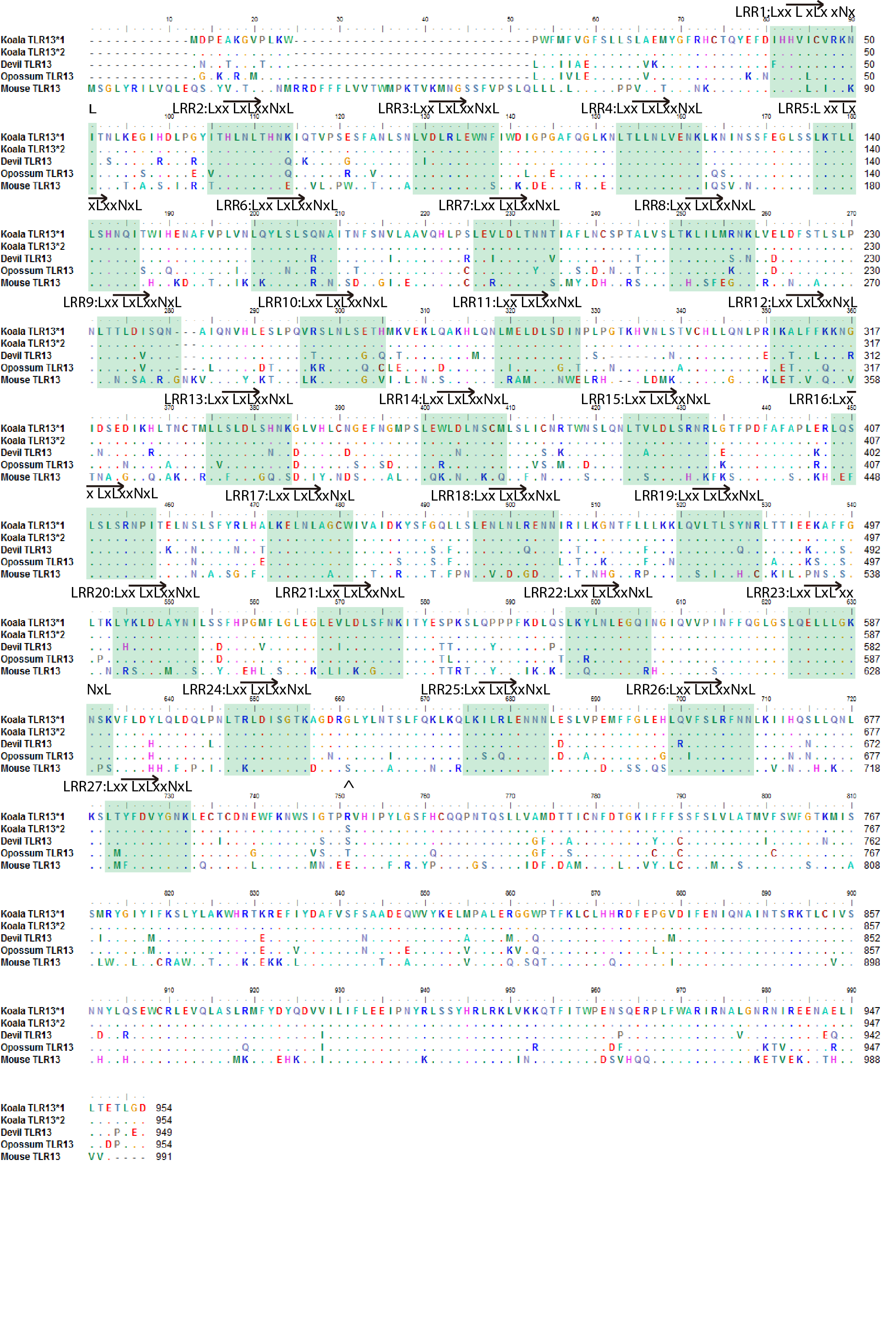

Supplement: S21 Fig — (TIF) [file pone.0121068.s021.tif]
